# Effects of low-level laser therapy on burning pain and quality of life in patients with burning mouth syndrome: a systematic review and meta-analysis

**DOI:** 10.1186/s12903-023-03441-w

**Published:** 2023-10-09

**Authors:** Chenghui Lu, Chenglong Yang, Xin Li, Guanhuan Du, Xuan Zhou, Wenhai Luo, Qing Du, Guoyao Tang

**Affiliations:** 1 Department of Oral Mucosal Diseases, The Affiliated Stomatological Hospital of Guilin Medical University, Guilin, 541004 China; 2 Department of Stomatology, Xinhua Hospital, Shanghai Jiao Tong University School of Medicine, 1665 Kongjiang Road, Shanghai, 200092 China; 3 Department of Rehabilitation, Xinhua Hospital, Shanghai Jiao Tong University School of Medicine, 1665 Kongjiang Road, Shanghai, 200092 China; 4 Department of Oral Mucosal Diseases, Shanghai Ninth People’s Hospital, Shanghai Jiao Tong University School of Medicine; College of Stomatology, Shanghai Jiao Tong University; National Center for Stomatology; National Clinical Research Center for Oral Diseases; Shanghai Key Laboratory of Stomatology, Shanghai, 200011 China

**Keywords:** Burning mouth syndrome, Low-level laser therapy, Burning pain, Quality of life, Negative emotions, Meta-analysis

## Abstract

**Background:**

Burning mouth syndrome (BMS) is a complex chronic pain disorder that significantly impairs patients' quality of life. Low-level laser therapy (LLLT) uses infrared or near-infrared light to produce analgesic, anti-inflammatory, and biological stimulation effects. The aim of this systematic review is to evaluate the effect of LLLT on burning pain, quality of life, and negative emotions in patients with BMS.

**Methods:**

The PubMed, Embase, Cumulative Index of Nursing and Allied Health Literature (CINAHL), Cochrane Library, Web of Science, and Scopus databases were searched up January 2023 to identify relevant articles. All randomized controlled trials that were published in English and examined the use of LLLT treatment for BMS were included. The methodological quality of the included trials was assessed using the Cochrane risk of bias tool for randomized controlled trials (RCTs). A meta-analysis was performed to evaluate burning pain, quality of life, and negative emotions. Sensitivity, subgroup, and funnel plot analyses were also carried out.

**Results:**

Fourteen RCTs involving a total of 550 patients with BMS met the inclusion criteria. The results showed that LLLT (measured by the Visual Analog Scale; SMD: -0.87, 95% CI: -1.29 to -0.45, *P* < 0.001) was more effective for reducing burning pain than placebo LLLT or clonazepam. LLLT improved quality of life (evaluated by the Oral Health Impact Profile-14; SMD: 0.01, 95% CI: -0.58 to 0.60, *P* = 0.97) and negative emotions (evaluated by the Hospital Anxiety and Depression Scale; SMD: -0.12, 95% CI: -0.54 to 0.30, *P* = 0.59), but these effects were not statistically significant.

**Conclusions:**

The meta-analysis revealed that LLLT may be an effective therapy for improving burning pain in patients with BMS, and producing a positive influence on quality of life and negative emotions. A long-term course of intervention, a larger sample size, and a multidisciplinary intervention design are urgently needed in future research.

**Trial registration:**

PROSPERO registration number: CRD42022308770.

**Supplementary Information:**

The online version contains supplementary material available at 10.1186/s12903-023-03441-w.

## Introduction

Burning mouth syndrome (BMS) is a complex chronic pain disorder that is often characterized by spontaneous, persistent, or recurrent burning pain or paraesthesia in the oral mucosa, with a prevalence ranging from 0.01% to 40% [1]. BMS is also regarded as a form of neuropathic pain. Evidence has suggested that neuroinflammation is involved in BMS and that proinflammatory cytokines and biomarkers, such as interleukin 6 (IL-6), tumor necrosis factor alpha (TNF-α), immunoglobulin A (IgA), and salivary cortisol, affect the nervous system, thus inducing the development of neuropathic pain and hyperalgesia [2–4]. This spontaneous, persistent, or recurrent burning pain causes an unpleasant sensory and emotional experience that tends to be positively correlated with the severity of BMS and significantly affects quality of life [5, 6]. Notably, this pain has been associated with an increased risk of suicide mortality, and studies have reported that BMS patients may have thoughts of and engage in behaviors related to suicide; therefore, BMS places a socioeconomic and medical burden on patients and health care systems [7, 8].

Current evidence supports the use of some BMS interventions, including pharmacological management (clonazepam) [9, 10], nonpharmacological management (low-level laser therapy (LLLT) [11, 12], and psychological interventions (cognitive behavioral therapy) [13, 14]. Of note, pharmacological management still exhibits large individual differences and may need long-term administration [9]. Additionally, the side effects of pharmacological management need to be carefully considered, such as nausea, vomiting, dizziness, and drowsiness [15], which limit patient adherence to the currently available pharmacotherapies. Cognitive behavioral therapy is also recommended for treatment-resistant BMS since BMS likely has a psychological origin [13]. However, dentists without a background in psychology cannot easily administer the intervention due to the high technical sensitivity [16]. Patients would like to consider treatment approaches that have low costs, few side effects and high executability, but there is no consensus regarding the optimal approach.

Noninvasive physical modalities (including LLLT) have been regarded as an important innovation in pain management (including among BMS patients) in recent years and are widely used in clinical settings, such as postherpetic neuralgia [17], oral mucositis [18], oral lichen planus [19] and neuropathic orofacial pain [20]. LLLT is also known as photobiomodulation therapy (PBMT) and uses infrared or near-infrared light to produce analgesic, anti-inflammatory, and biological stimulation effects; LLLT is recommended as a complementary treatment option when pharmacotherapy alone is not sufficient [21]. Recent findings on the effects LLLT on pain relief among patients with BMS remain controversial due to different intervention protocols and parameters [22, 23]; therefore, a systematic quantitative analysis is necessary. Some studies have shown that longer wavelengths and higher irradiance could reduce symptoms in patients with BMS and have sustained and lasting effects [11, 12, 24, 25], while other studies have demonstrated that shorter wavelengths and lower irradiance could also reduce burning symptoms [23, 26, 27]. The main purpose of this meta-analysis was to systematically and quantitatively review the effects of LLLT on burning pain, quality of life, and negative emotions in patients with BMS. The relationship between intervention protocols and parameters and the efficacy of LLLT was also analyzed.

## Materials and methods

### Protocol and registration

This meta-analysis was prospectively registered in the PROSPERO database (https://www.crd.york.ac.uk/PROSPERO) with registration number CRD 42022308770. The Preferred Reporting Items for Systematic Reviews and Meta-Analyses (PRISMA) guidelines were followed to conduct this systematic review [28].

### Literature search and selection criteria

The following electronic databases were searched for studies published up to January 2023: PubMed, Embase, Cumulative Index of Nursing and Allied Health Literature (CINAHL), the Cochrane Library, Web of Science, and Scopus. The keywords used to identify LLLT were 'low-level laser therapy' and 'LLLT', while the keywords used to identify BMS were 'burning mouth syndrome' and 'BMS ∗ '. The reference lists of the included articles were also searched to identify additional studies. A comprehensive search strategy (Additional file 1) was developed to search for studies that evaluated the use of LLLT for the treatment of BMS.

Studies were considered eligible if they met the prespecified study criteria and investigated the effectiveness of LLLT for the treatment of BMS, irrespective of sex, age, and country (Table 1).

**Table 1:** PICOS criteria for study inclusion

Parameter	Inclusion criteria	Exclusion criteria
Population	Patients with a diagnosis of BMS according to the International Classification of Headache Disorders-3 (ICHD-3) [29]: patients presenting symptoms of oral burning or pain lasting more than 2 h per day for more than 3 months	Any local or systemic factors that could produce the symptoms of oral burning pain, such as oral infections, oral lichen planus, or oral candidiasis
Intervention	LLLT (600–1100 nm) was delivered directly to the site of pain; no limitations were placed on exposure duration or distance	
Comparator	No treatment or other treatments	
Outcomes	Primary outcome: 1) Burning pain, measured using the Visual Analog Scale (VAS) Secondary outcomes: 1) Oral health-related quality of life, assessed by the Oral Health Impact Profile-14 (OHIP-14); 2) Negative emotions, measured using the Hospital Anxiety and Depression Scale (HADS); 3) Other relevant outcomes and serious adverse events	
Study design	1) Randomized controlled trials; 2) Published in English	1) Observational studies; 2) Non-randomized controlled trials; 3) Other types of studies

### Data extraction and quality assessment

Full-text articles that were deemed eligible or potentially eligible for inclusion were retrieved and independently screened by three reviewers (LCH, YCL, and LX). Disagreements were resolved via consensus. LCH independently extracted data using a standardized data extraction form, which was double-checked by DGH. The following data were extracted: study design, inclusion criteria, participant demographics (age, sex, number of participants (% women), and underlying conditions), disease characteristics (number of burning sites), intervention details (wavelength, source, intensity, duration of light, the distance of light exposure from the oral mucosa, exposure dose, and any other adjunctive or subsequent interventions), comparison details and outcome data (burning pain and quality of life). Furthermore, the original investigators were contacted to provide detailed information regarding any unreported data.

Three independent raters (LCH, YCL, and LX) assessed the methodological quality of the studies using the Cochrane Risk of Bias (RoB) tool for RCTs [30], and any disagreement was resolved through discussion or by consulting another reviewer (DGH). There are five domains assessed by the RoB 2.0: the randomization process, deviations from the intended intervention, missing outcome data, measurement of the outcome, and selection of the reported outcomes. For missing outcome data in individual studies, we defined a low risk of bias as a loss to follow-up less than 10% and a difference of less than 5% in missing data between intervention and control groups. Funnel plots were constructed to assess publication bias [31]. In addition, we assessed the quality of the evidence using the Grading of Recommendations, Assessment, Development, and Evaluation (GRADE) criteria [32] categorized the quality into one of four levels (high, moderate, low, or very low). Additional file 2 shows the GRADE assessments.

### Statistical analysis and data synthesis

All analyses were performed using RevMan (version 5.4.1) or Stata (version 16.0). The median, interquartile range, and sample size in each trial were acquired to estimate the mean and standard deviation (SD) for each study, and simple and basic inequalities and approximations were used as necessary [33]. Data, such as the mean differences in burning pain, quality of life, and anxiety before and after interventions, were converted to the mean ± SD [34]. The results are presented as the weighted mean difference (WMD) or standardized mean difference (SMD). Ninety-five percent confidence intervals (CIs) were used to evaluate the effect size for each study. The *I*^*2*^ statistic was used to assess heterogeneity between studies. Data were combined by a fixed effect model when *I*^*2*^ < 50%. Otherwise, a random effects model was used. *I*^*2*^ values of less than 25% indicated low heterogeneity, value from 26–50% indicated moderate heterogeneity, and values greater than 50% indicated high heterogeneity [35]. Furthermore, given the high degree of heterogeneity of the true differences in the effect sizes, we ran a meta-regression to regress the burning pain upon risk of bias (high, low, unclear risk of bias), publication year (< 5 years, > or = 5 years), laser wavelength (> 780 nm, 600–700 nm), irradiance (> 50 mW/cm^2^, < or = 50 mW/cm^2^), intervention duration (< or = 4 weeks intervention, > 4 weeks intervention), and intervention frequency (< or = 2 times intervention per week, > 2 times intervention per week). Subgroup analysis or sensitivity analysis were performed to determine the sources of heterogeneity. Differences were deemed significant if the P value was < 0.05 between the two groups.

## Results

### Study identification and selection

**Fig. 1:**
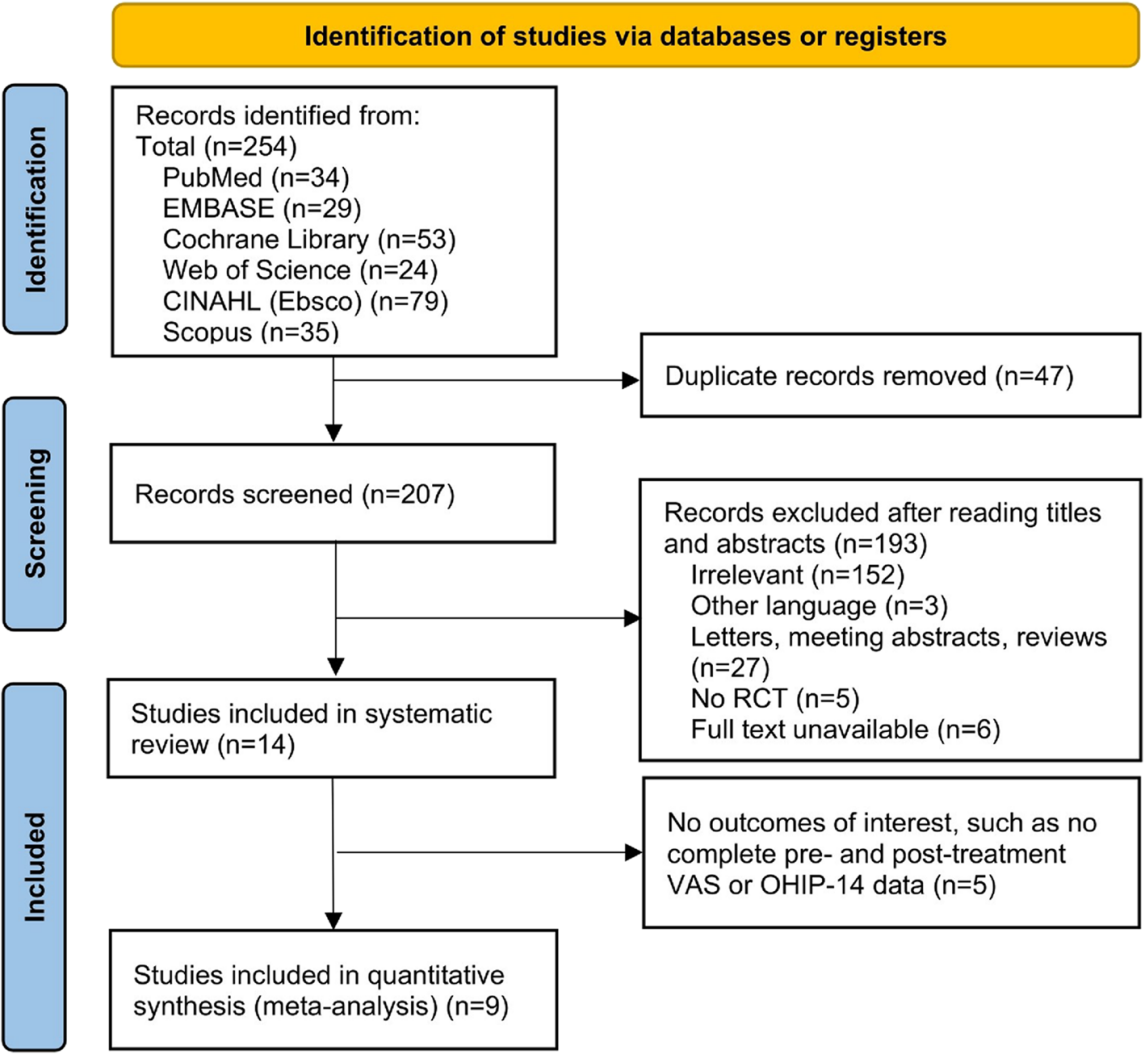
PRISMA flowchart of the studies included in this review

After carefully reviewing 254 references and 222 full-text articles from six databases, we ultimately included fourteen studies that met the inclusion criteria, involving 550 patients with valid outcome data. Fourteen articles examined the effect of LLLT on BMS. Nine of these studies were included in the quantitative analysis, with 229 BMS patients and 215 control patients. Figure 1 illustrates the PRISMA flowchart.

### Description of the included studies

The demographic and baseline characteristics of the included trials and their participants are summarized in Table 2. The included studies were published between 2010 and 2021, with an overall dropout rate of 2.18% (*n* = 12). Of the 550 participants, 87.10% (*n* = 479) were women, with a male-to-female ratio of approximately 7 to 1. The mean age of the participants was 61.12 ± 8.99 years, with a mean disease duration of 23.86 ± 18.05 months (range: 2 to 192 months). The tongue accounted for up to 80% of affected sites, followed by the gums, lips, and hard palate.

**Table 2:** Baseline demographic and clinical characteristics of the study participants

	Author (year)	Study design	Country	Participants (% women)	Average age (year, Mean ± SD) (range)	Average disease duration (month, Mean ± SD) (range)	Most common site (%)	Outcome measures and overall results (positive + /negative-)	Serious adverse events	Dropout rate	Time points
1	Pezelj-Ribaric et al., 2013 [36]	Randomized controlled trial	Croatia	(1) LG: 20; (2) CG: 20 (67.5%)	(1) LG: 60.2 ± 6.3; (2) CG: 61.1 ± 2.2	Not mentioned	Tongue	(1) TNF-α and IL-6 + (2) Pain/burning (VAS) +	Not mentioned	0%	Baseline 4 weeks
2	Spanemberg et al., 2015 [37]	Randomized controlled trial	Spain	(1) LG1: 20 (2) LG2: 20 (3) RLG: 19 (4) CG: 19 (85.9%)	62.82 ± 7.54 (45–79)	6 or above (up to 30 years)	Tongue (up to 90%) Lips (up to 50%) Palate (up to 42.1%) Other sides (up to 20%)	(1) Pain/burning (VAS/VNS) + (2) Oral health-related quality of life (OHIP-14) +	None	0%	Baseline 10 weeks 8-week follow-up
3	Arbabi-Kalati et al., 2015 [26]	Randomized controlled trial	Iran	LG: 10 CG: 10 (100%)	(1) LG: 47.2 ± 5.3 (2) CG: 46.6 ± 4.6	(1) LG: 13.4 ± 7.4 (6–30) (2) CG: 15.5 ± 0.1 (6–36)	Not mentioned	(1) Pain/burning (NRS) + (2) Oral health-related quality of life (OHIP-14) +	None	0%	Baseline 2 weeks
4	Sugaya et al., 2016 [38]	Randomized controlled trial	Brazil	(1) LG: 15 (2) CG: 15 (91.3%)	(1) LG: 59.3 (29–83) (2) CG: 62.7 (53–81)	(1) 25.5 (6–192) (2) 39.6 (6–180)	Tongue Lower lip Upper lip Buccal mucosa Mandibular ridge Palate Mandibular gingiva	Pain/burning (VAS) +	None	23.33%	Baseline 2 weeks 7, 14, 30, 60, and 90-day follow-ups
5	Valenzuela et al., 2016 [25]	Randomized controlled trial	Spain	(1) LG: 16 (2) LG inf: 16 (3) CG: 12 (93.2%)	65.5 ± 10.6 (33–88)	6 or above	Not mentioned	(1) Pain/burning (VAS) + (2) Oral health-related quality of life (OHIP-14) + (3) Xerostomia severity (Xerostomia Inventory) - (1) Anxiety and depression (HADS) - (2) Overall patient satisfaction (PGI-I) -	Not mentioned	0%	Baseline 4 weeks
6	Arduino et al., 2016 [22]	Randomized controlled trial	Italy	(1) LG: 18 (2) CG: 15 (75.8%)	67.12 ± 8.58	6 or above	Not mentioned	(1) Pain/burning (VAS + /McGill + /PPI +) (2) Oral health-related quality of life (OHIP-14) + (3) Salivary flow - (4) Anxiety and depression (HADS) + , (GDS) +	None	0%	Baseline 5 weeks 3, 8, and 12-month follow-ups
7	Sikora et al., 2018 [24]	Randomized controlled trial	Croatia	(1) LG: 22 (2) CG: 22 (97.7%)	67.56 (56–83)	Not mentioned	Not mentioned	(1) Oral health-related quality of life (OHIP-14) + (2) Pain/burning (VAS) +	Not mentioned	0%	Baseline 2 weeks
8	Spanemberg et al., 2019 [39]	Randomized controlled trial	Spain	(1) LG: 12 (1) CG: 9 (95.2%)	(1) LG: 66.3 ± 7.52 (2) CG: 66.2 ± 6.31 (61–81)	57.8 (8–130)	Tongue (61.9%) Lips (52.4%) Palate (42.9%) Other sides (28.6%)	(1) Pain/burning (VAS) + (1) Anxiety and depression (HADS) -	None	0%	Baseline 8 weeks 2-month follow-up
9	Bardellini et al., 2019 [40]	Randomized controlled trial	Italy	(1) LG: 45 (2) CG: 45 (100%)	(1) LG: 59.76 ± 9.51 (39–74) (2) CG: 60.86 ± 10.02 (41–77)	6 or above	Tongue (76.5%) Lips (18.8%) Buccal mucosa (44.7%) Other sides (9.4%)	(1) Pain/burning (VAS) + (2) Oral health-related quality of life (OHIP-14) +	Not mentioned	5.6%	Baseline 10 weeks 1-month follow-up
10	de Pedro et al., 2020 [20]	Randomized controlled trial	Spain	(1) LG: 10 (2) CG: 10 (80%)	(1) LG: 60.30 ± 15.19 (2) CG: 67.60 ± 10.68	Not mentioned	Tongue (100%) Buccal mucosa (45%) Lips (30%) Hard palate (10%)	(1) Pain/burning (VAS/McGill) + (2) Oral health-related quality of life (OHIP-14) + (3) General health status (SF-36) + (4) Drowsiness/sleepiness (ESS) + (5) Anxiety and depression (SCL 90-R) +	None	0%	Baseline 5 weeks 1 and 4-month follow-ups
11	Skrinjar et al., 2020 [23]	Randomized controlled trial	Croatia	(1) LG: 12 (2) CG: 11 (86.9%)	(1) LG: 61.5 (47–70) (2) CG: 62 (50–69)	3 or above	Tongue Lip Hard palate	(1) Pain/burning (VAS) + (2) Salivary cortisol level +	None	0%	Baseline 2 weeks
12	Barbosa et al., 2020 [27]	Randomized controlled trial	Brazil	(1) LG: 10 (2) CG: 5 (60%)	45 (40–52)	12 (4–24)	Tongue (66.7%) Lips (26.7%) Palate (20%) Cheek mucosa (20%) Alveolar ridge (13.3%)	(1) Salivary flow + (2) TNF-α - (3) Pain/burning (VAS) +	None	0%	Baseline 4 weeks
13	Scardina et al., 2020 [41]	Randomized controlled trial	Italy	(1) LG: 20; (2) CG: 20 (100%)	62.06 ± 3.1	Not mentioned	Upper labial mucosa; Buccal mucosa Dorsal lingual surface Lower labial mucosa	(1) Pain/burning (VAS/NRS) + (2) Capillary microcirculation (Oral videocapillaroscopy examination) +	None	0%	Baseline 4 weeks 2-month follow-up
14	Sun et al., 2021 [12]	Randomized controlled trial	China	(1) LG: 21 (2) CG: 21 (80.9%)	(1) LG: 56.19 (2) CG: 47 (19–71)	(1) LG: 11.8 (2) CG: 7.00 (2–60)	Tongue (100%)	(1) Pain/burning (VAS) + (2) Numbness (VAS) + (3) Alter taste (VAS) -	None	0%	Baseline 4 weeks

*AG* Acupuncture group, *CG* Control group, *ESS* Epworth Sleepiness Scale, *GDS* Geriatric Depression Scale, *HADS* Hospital Anxiety and Depression Scale, *LG* laser group, *LG inf* Infrared laser group, *IL-6* Interleukin- 6, *McGill* McGill Pain Questionnaire, *NRS* Numeric Rating Scale, *OHIP-14* Oral Health Impact Profile-14, *PGI-I* Patient Global Impression of Improvement, *PPI* Present pain intensity, *RLG* Red laser group, *RG* Repetitive transcranial magnetic stimulation group, *SF-36* Short Form 36 Health Survey, *SCL-90R* Symptom Checklist 90, *TNF-α* Tumor necrosis factor-α, *VAS* Visual Analog Scale, *VNS* Visual numeric scale

The detailed LLLT methods and control protocols are summarized in Table 3. Nine of the fifteen studies employed GaAlAs lasers [22–26, 36–39], while the others used Nd:YAG lasers [12], K-laser Cube 3 [40], BioLase Epic10 [41], Fox diode laser [11], and class 3B visible low-level laser [27]. Of the included studies, the parameters of LLLT application were heterogeneous, including laser wavelength (range: 630 to 1064 nm), power (range: 30 mW to 4 W), and irradiance (range: 0.003 to 4 W/cm^2^). The wavelength used in nine of the fifteen studies was > 780 nm [12, 20, 22, 24, 25, 37–39, 41], and four studies used wavelength of 600–700 nm [23, 26, 27, 36]. Bardellini et al. [40] used a continuous spectral range (660–970 nm). The control group mostly received placebo LLLT (sham/inactive laser) [12, 20, 23–26, 36–41]; ALA [27] and clonazepam [22] were administered in some studies. A total of seven trials reported follow-up data: six of these studies had follow-up durations between one and four months [20, 37–41], and one study reported a follow-up of 12 months [22]. The mean total treatment duration of the fifteen trials was 4.64 ± 2.79 weeks (median: 4 weeks; range: 2 to 10 weeks), and the mean follow-up period for seven trials was 16.80 ± 18.80 weeks (median: 8 weeks; range: 4 weeks to 12 months).

**Table 3:** Low-level laser therapy and control interventions in the included trials

	Author (year)	Physical therapy in the intervention group	Control group intervention	Frequency	Time points
1	Pezelj-Ribaric et al., 2013 [36]	GaAlAs, 685 nm, 30 mW, 3.0 J/cm^2^, 100 s/point, irradiation area: 1 cm^2^, tip diameter: 2 mm	Inactive/placebo laser: the same time and the same points, but without power	5 times per week	Baseline 4 weeks
2	Spanemberg et al., 2015 [37]	(1) LG1: GaAlAs, 830 nm, 100 mW, 5 J/point, 176 J/cm^2^, 3.57 W/cm^2^, 50 s/point (2) LG2: GaAlAs, 830 nm, 100 mW, 5 J/point, 176 J/cm^2^, 3.57 W/cm^2^, 50 s/point (3) RLG: 685 nm, 35 mW, 2 J/point, 72 J/cm^2^, 1.25 W/cm^2^, 58 s/point	Sham LLLT: searching for similarities to the IR3 W and red laser groups; however, the tool received a plastic tip with a rubber interior that blocked radiation emission	(1) LG1: once per week (2) LG2: 3 times per week (3) RLG: 3 times per week (4) CG: 3 times per week	Baseline 10 weeks 8-week follow-up
3	Arbabi-Kalati et al., 2015 [26]	GaAlAs, 630 nm, 30 mW, 1 J/cm^2^, 10 s/point. Laser application points: 10 areas on the oral mucosa, 2 areas on the buccal mucosa on each side, 2 areas on the tongue, 2 areas on the floor of the mouth, 1 area on the soft palate, and 1 area on the hard palate	Inactive/placebo laser: the same period, the same areas but the laser was silent	twice per week	Baseline 2 weeks
4	Sugaya et al., 2016 [38]	GaAlAs, 790 nm, 120 mW, 4 W/cm^2^, 6 J/cm^2^, 50 s/point, irradiation area: 0.03 cm^2^	Inactive/placebo laser: the same procedures but the device turned off	twice per week	Baseline 2 weeks 7, 14, 30, 60, and 90-day follow-ups
5	Valenzuela et al., 2016 [25]	(1) LG: GaAlAs, 815 nm, 1 W, 4 s/point, 133.3 J/cm^2^; irradiation area: 0.03 cm^2^ (2) LG inf: GaAlAs, 815 nm inf, 1 W, 6 s/point, 200 J/cm^2^ irradiation area: 0.03 cm^2^	Sham LLLT: the same procedure but the laser turned off	once per week	Baseline 4 weeks
6	Arduino et al., 2016 [22]	GaAlAs, 980 nm, 300 mW, 1 W/cm^2^, 10 J/cm^2^, 10 s/point, irradiation distance: 2 mm, area: 0.28 cm^2^, tip diameter: 6 mm. All the mucosal burning sites were irradiated	Clonazepam: suck half a tablet of 2 mg of clonazepam and hold their saliva near the pain sites in the mouth without swallowing for 3 min and then spit. This protocol has to be repeated three times a day for 21 days	5 times per week	Baseline 5 weeks 3, 8, and 12-month follow-ups
7	Sikora et al., 2018 [24]	GaAlAs, 830 nm, 100 mW, 12 J/cm^2^, irradiation distance: 5 mm, area: 1 cm^2^; switched on: 800 ms, switched off: 1 ms. Laser application points: the site in the mouth where burning symptoms	Sham laser: LLLT switched off	5 times per week	Baseline 2 weeks
8	Spanemberg et al., 2019 [39]	GaAlAs, 808 ± 5 nm, 200 mW, 1.97 W/cm^2^, 3 J/point, 15 s/point, irradiation area: 0.088 cm^2^. Laser application points: the tip of the tongue: 3 points; lateral border of the tongue: 4 points; dorsal surface of the tongue: 10 points; buccal mucosa: 8 points; labial mucosa: 5 points, hard palate: 8 points, soft palate: 3 points; gingiva or alveolar mucosa: 3 points by sextant	Inactive/placebo laser: the same protocol but the laser was deactivated. Neither the patient nor the researcher knew if the laser was activated or not	once per 2 weeks	Baseline 8 weeks 2-month follow-up
9	Bardellini et al., 2019 [40]	K laser Cube 3, 660–970 nm, 3.2 W, 1–20 000 Hz, irradiation area: 1 cm^2^. The most painful areas in the oral cavity were irradiated	Inactive/placebo laser: the device was turned on but the handpiece did not work	once per week	Baseline 10 weeks 1-month follow-up
10	de Pedro et al., 2020 [20]	Diode Laser Fox, 810 nm, 0.6 W, 1.2 W/cm^2^, 6 J, 12 J/cm^2^, 10 s/point, irradiation area: 0.5 cm^2^. Laser application points: 56 points (3 in the vestibular mucosa of the 4 quadrants, 4 in each lip mucosa, 6 in each of the two buccal mucosae, 6 in the hard palate, 4 on each lateral edge of the tongue, 6 in the dorsum of the tongue and 4 sublingual points) with a distance in between of 2 mm	Inactive/placebo laser: the same 56 points, 10 s per point, and the same number of sessions but the device turned off	twice per week	Baseline 5 weeks 1 and 4-month follow-ups
11	Skrinjar et al., 2020 [23]	GaAlAs, 685 nm, 30 mW, 0.003 W/cm^2^, cumulative dose: 60 J/cm^2^; dose: 2 J/cm^2^, 5.20 Hz; irradiation distance: 5 mm, area: 3 cm^2^	Inactive/placebo laser: the same treatment protocol but LLLT was done with an inactive laser probe which was only emitting the audio signal	5 times per week	Baseline 2 weeks
12	Barbosa et al., 2020 [27]	Visible low-level class 3B laser, 660 nm, 30 mW, 3 J/cm^2^, 10 s/point, irradiation distance: 10 mm, tip diameter: 3 mm	ALA: treated for 30 days with 600 mg ALA (3 tablets of 200 mg per day after meals) and 150 mg ranitidine for gastric protection (one tablet in the morning)	once per day	Baseline 4 weeks
13	Scardina et al., 2020 [41]	BioLase Epic10, 805 nm, 4 W power, 60 mW continuous wave, 1200 J, 50 J/cm^2^, 166.7 mW/cm^2^, 300 s, irradiation distance: 40 mm. Laser application points: the upper labial mucosa, buccal mucosa, dorsal lingual surface, and lower labial mucosa	Inactive/placebo laser: the same sessions, the only difference was the non-emission of the laser	twice per week	Baseline 4 weeks 2-month follow-up
14	Sun et al., 2021 [12]	Nd: YAG laser, 1064 nm; 100 mW, 3 J/cm^2^, 10 Hz, 30 s/point; irradiation distance: 6 mm, area: 1 cm^2^. Laser application points: the tongue was divided into 17 treatment regions and only areas of the tongue reported as symptomatic were irradiated	Inactive/placebo laser: instrument switched off	once per week	Baseline 4 weeks

*LG* Laser group, *LG inf* Infrared laser group, *RLG* Red laser group

### Quality assessment

**Fig. 2:**
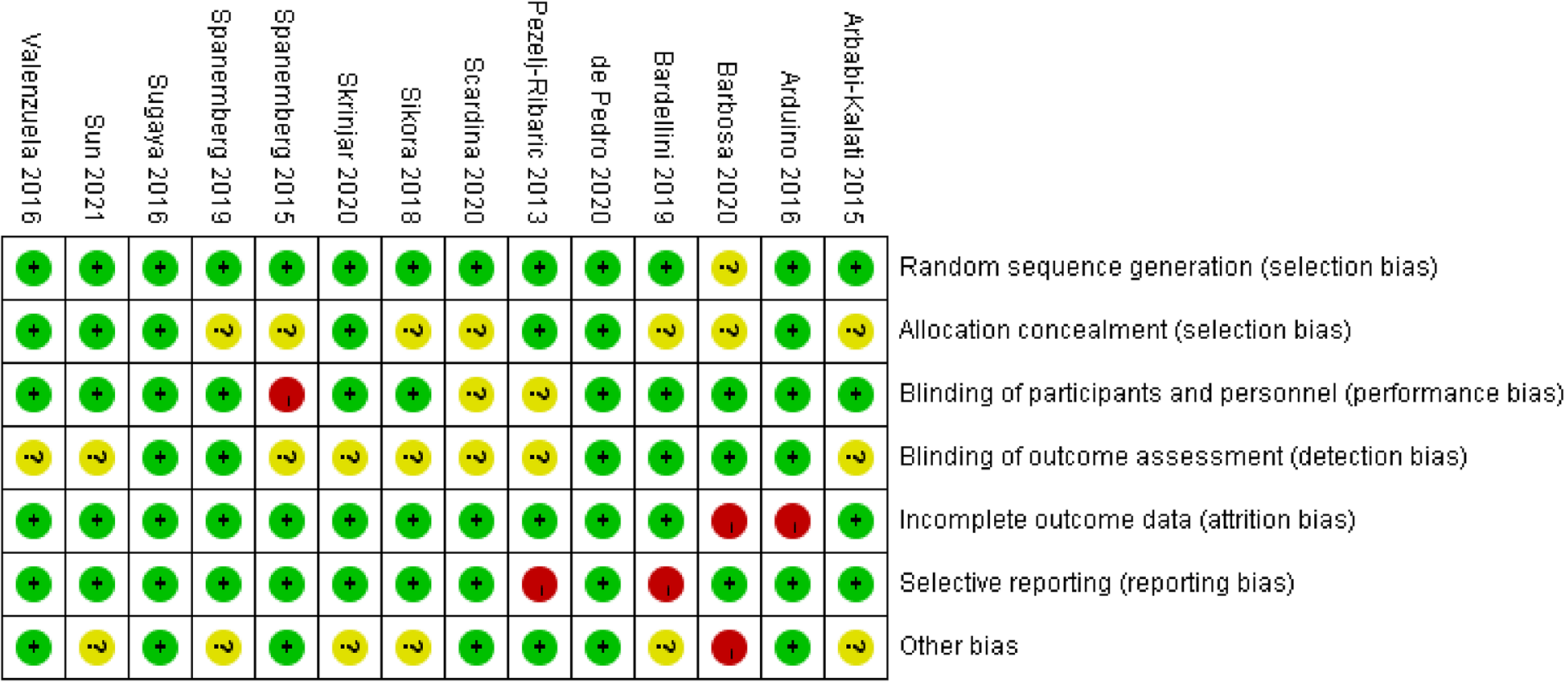
Risk of bias summary. The risk of each bias in the included studies is shown (+ , ?, and—indicate low, uncertain, and high bias, respectively)

**Fig. 3:**
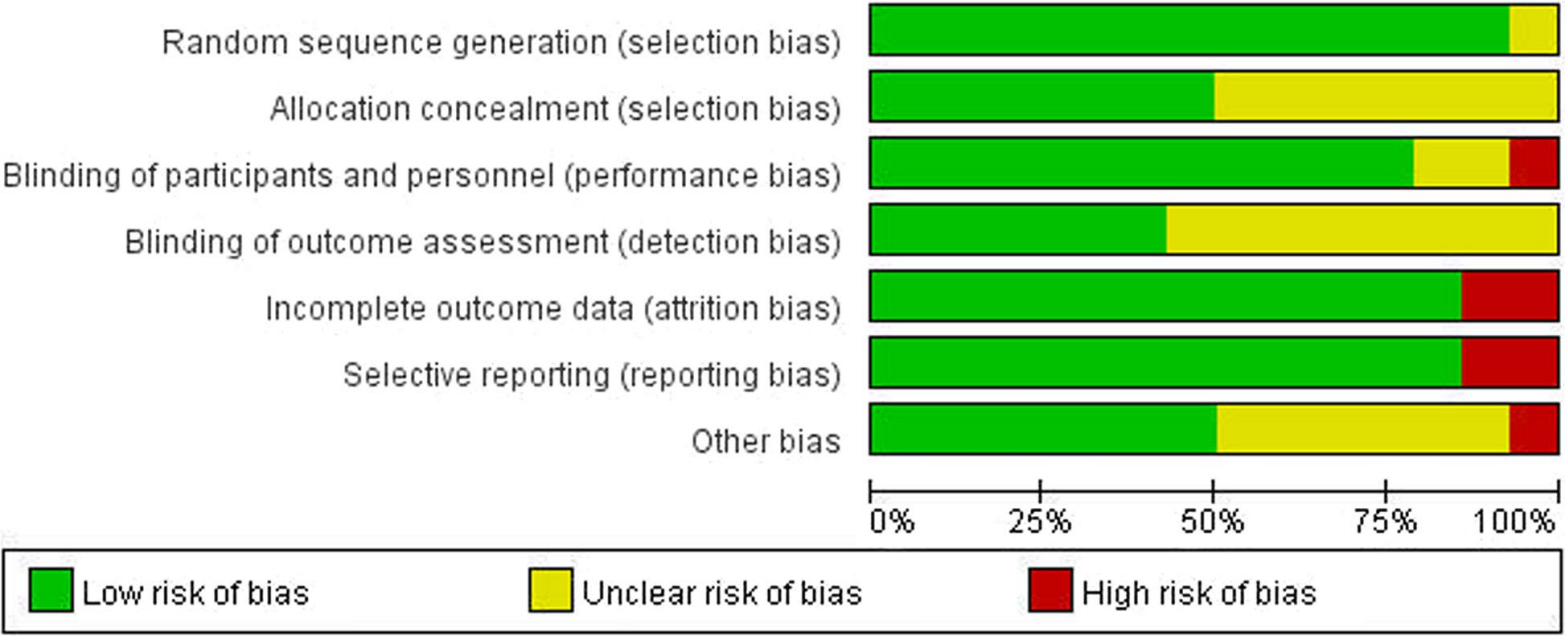
Risk of bias graph

According to the Cochrane Risk of Bias tool, two RCTs had a low risk of bias [20, 38], seven RCTs had an unclear risk of bias [12, 23–26, 39, 41] and five RCTs had a high risk of bias [22, 27, 36, 37, 40]. Only two of the fourteen trials reported the clinical identifier and were considered rigorous RCTs [20, 38]. Four studies detailed the random assignment method and were double-blinded [23, 39–41]. Three studies were single-blinded [20, 24, 25]. Three studies used randomization but did not describe the randomization method in detail [26, 27, 36]. Details of the risk of bias assessments are given in Figs. 2, 3.

### Outcome measurements

#### Primary outcome (burning pain)

**Fig. 4:**
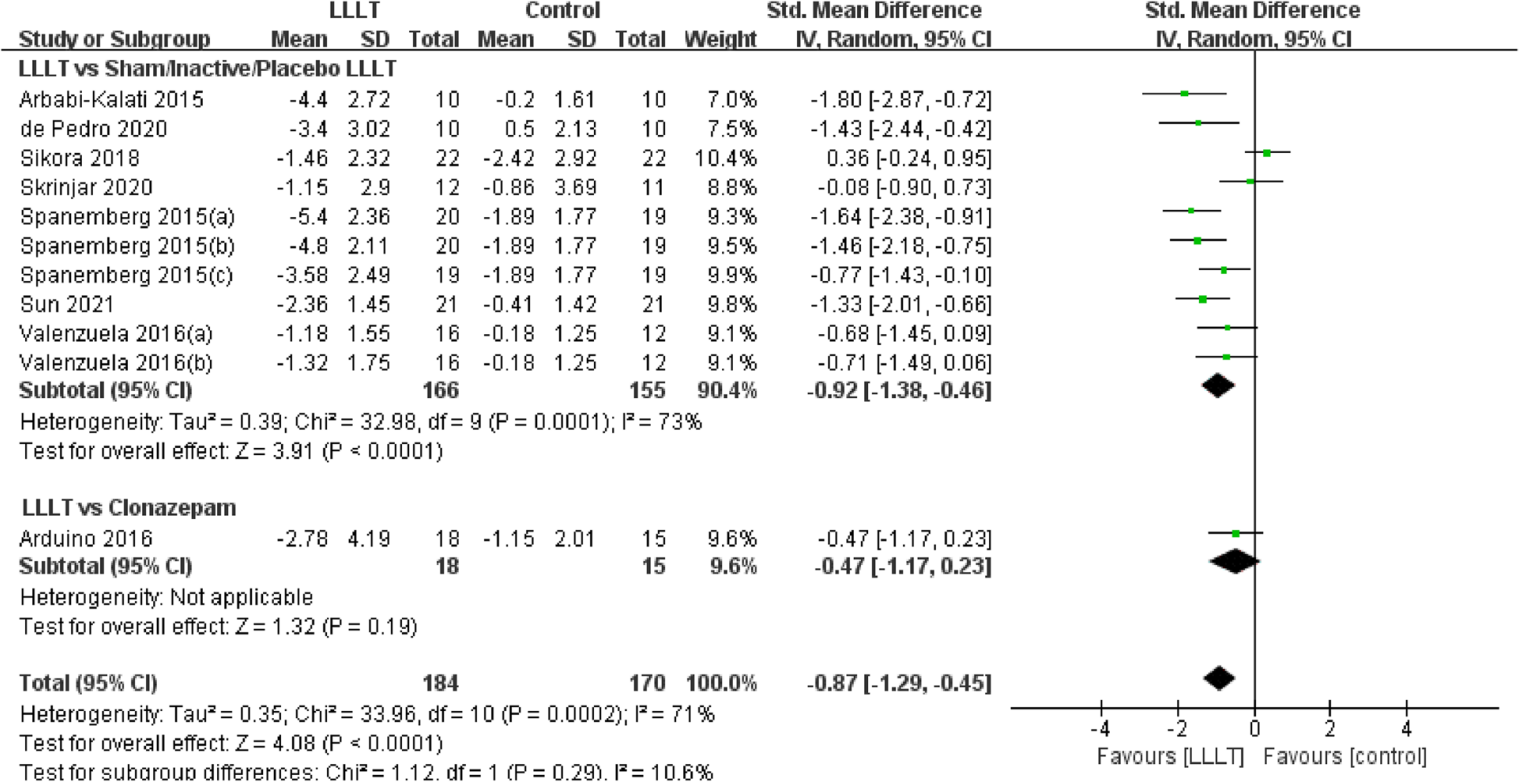
Forest plot and meta-analysis of changes in pain intensity. Subgroup analysis with different intervention methods as moderators

**Fig. 5:**
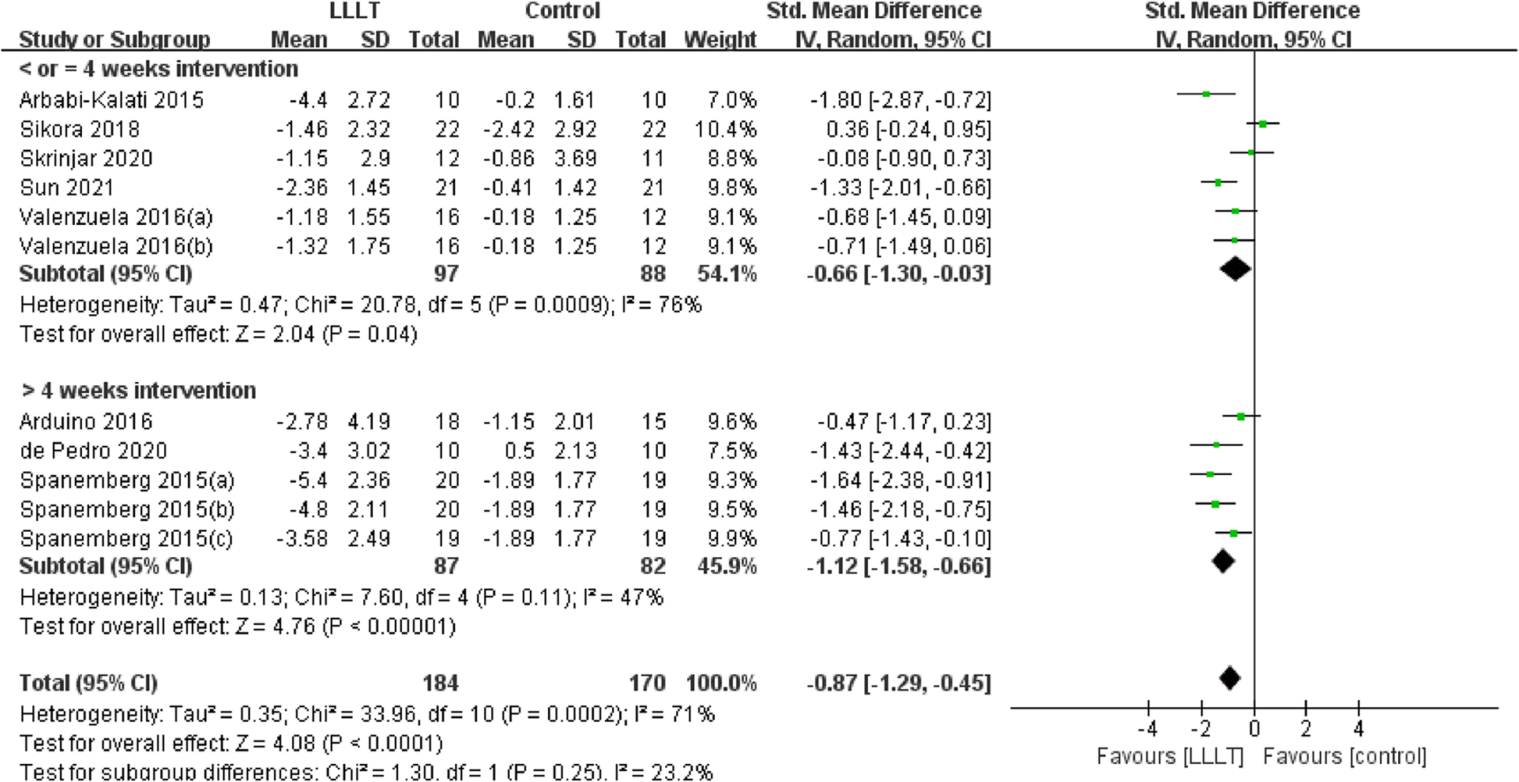
Forest plot and meta-analysis of changes in pain intensity. Subgroup analysis with different intervention durations as moderators

**Fig. 6:**
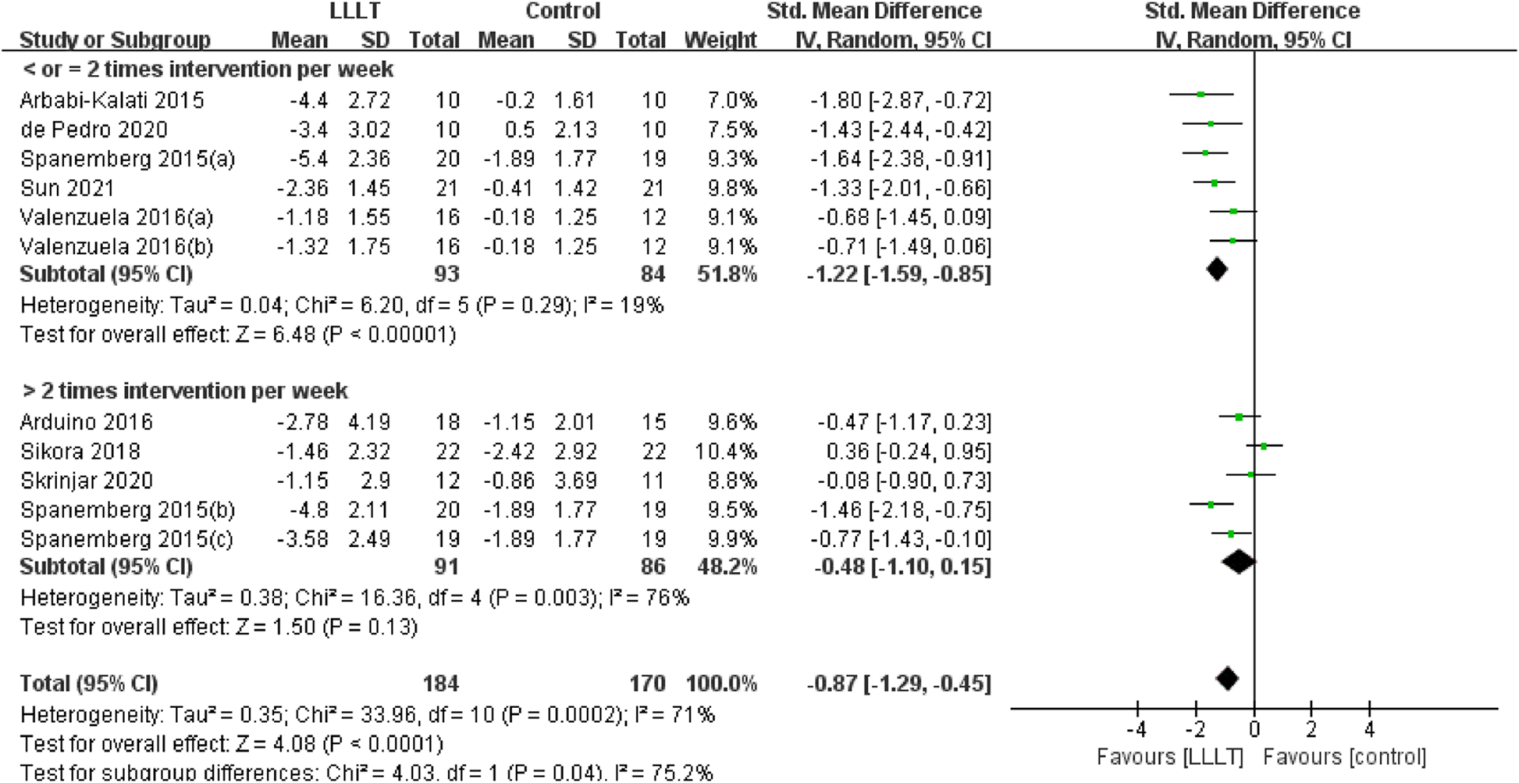
Forest plot and meta-analysis of changes in pain intensity. Subgroup analysis with different intervention frequency as moderators

**Fig. 7:**
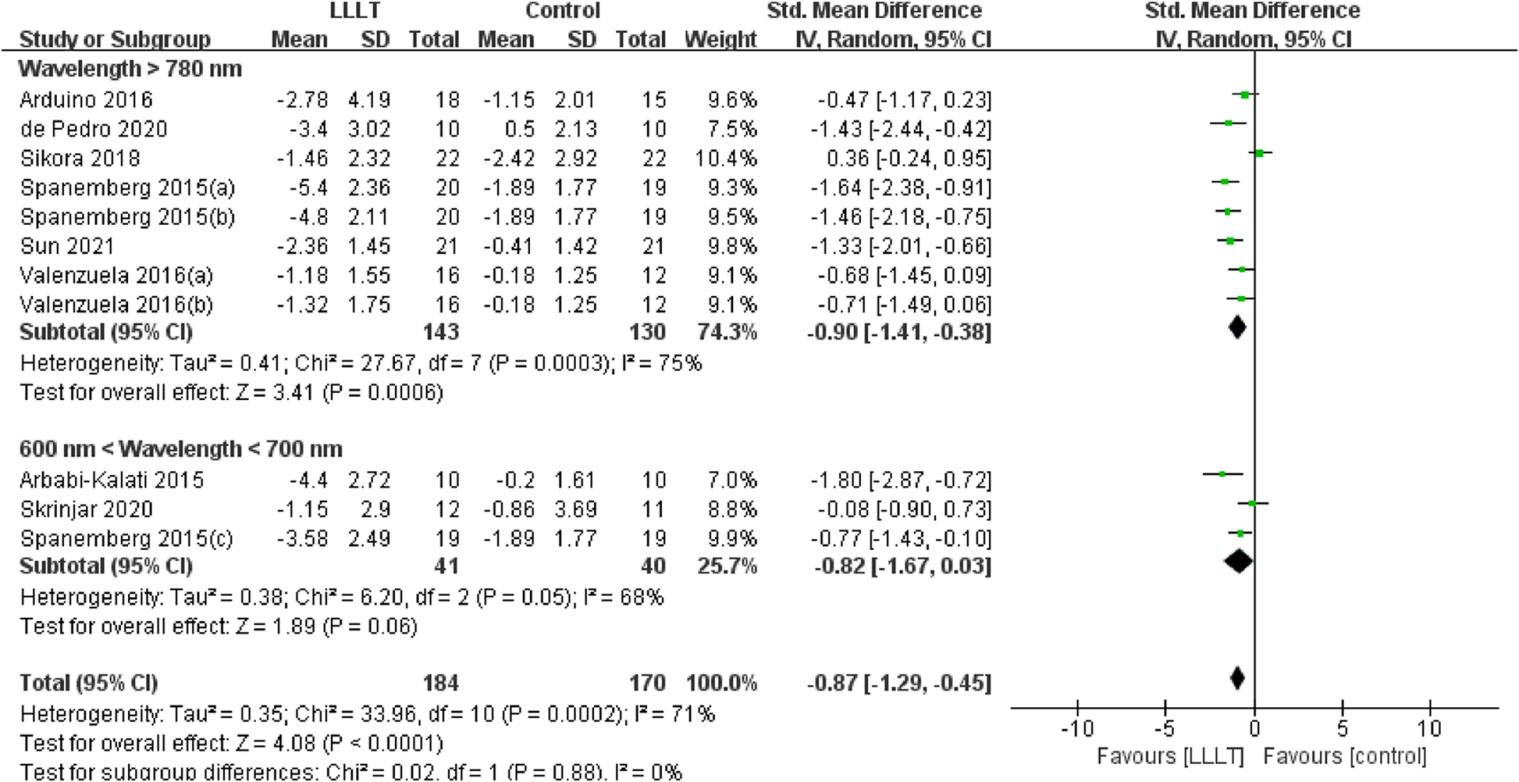
Forest plot and meta-analysis of changes in pain intensity. Subgroup analysis with different wavelengths as moderators

**Fig. 8:**
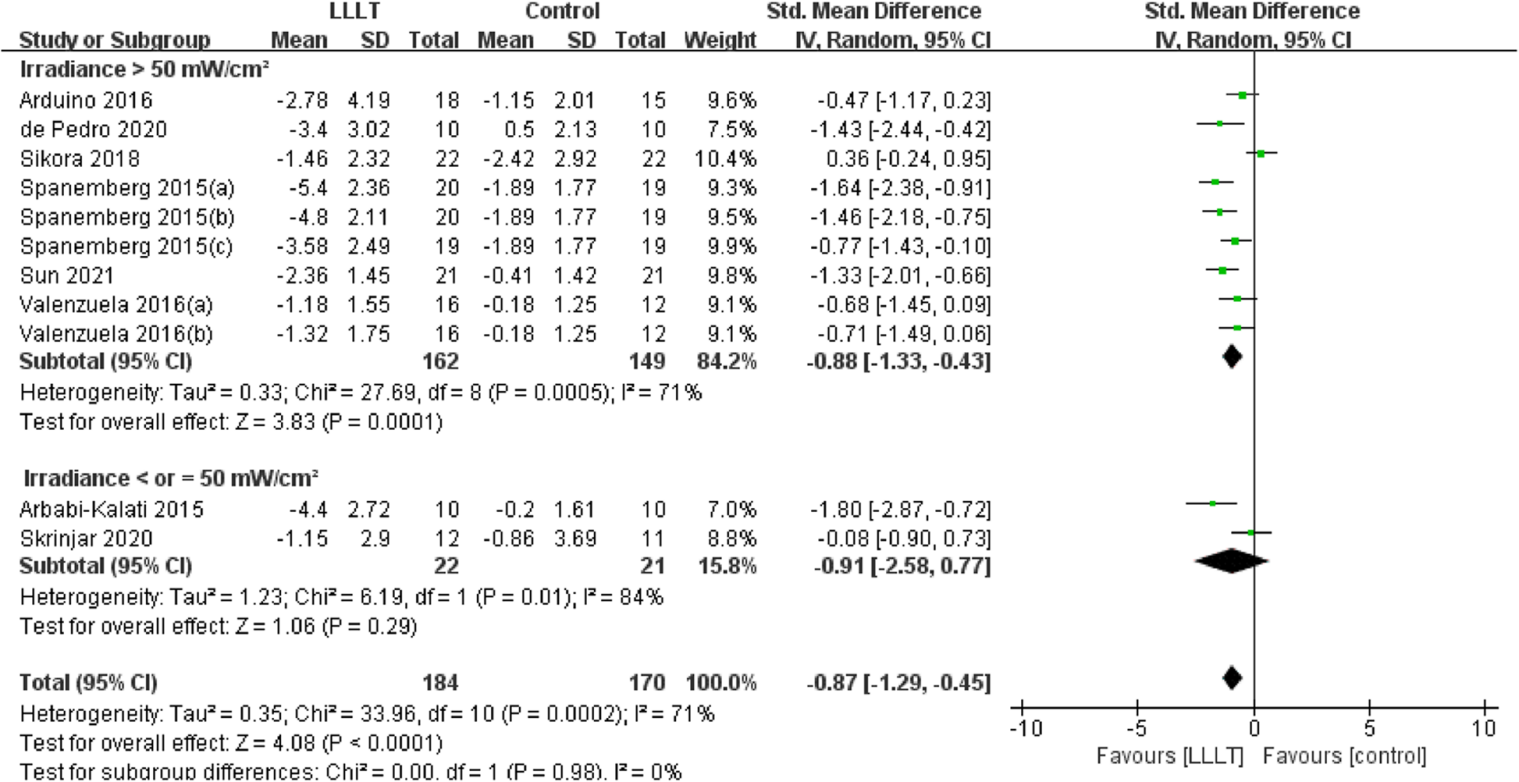
Forest plot and meta-analysis of changes in pain intensity. Subgroup analysis with different irradiances as moderators

Changes in burning pain (measured by Visual Analogue Scale) occurred in eight RCTs [12, 20, 22–26, 37] involving 354 participants (SMD: -0.87, 95% CI: -1.29 to -0.45, *P* < 0.001; *I*^*2*^ = 71%). After analyzing the effects of LLLT on burning pain intensity, the pooled analysis showed that LLLT was significantly more effective than sham LLLT in reducing pain intensity (SMD: -0.92, 95% CI: -1.38 to -0.46, *P* < 0.001;* I*^*2*^ = 73%) and slightly more effective than clonazepam (SMD: -0.47, 95% CI: -1.17 to 0.23, *P* = 0.19), with high heterogeneity (Fig. 4). Subgroup analysis was used to verify whether different factors would affect the changes in burning pain intensity. The results showed that LLLT reduced burning pain intensity when the intervention duration was > 4 weeks (SMD: -1.12, 95% CI: -1.58 to -0.66, *P* < 0.001; *I*^*2*^ = 47%; Fig. 5) and when the intervention frequency was < or = 2 times per week (SMD: -1.22, 95% CI: -1.59 to -0.85, *P* < 0.001; *I*^*2*^ = 19%; Fig. 6). This finding indicated that an intervention lasting at least four weeks and performed once or twice per week was an effective treatment option. However, efficacy did not significantly differ by wavelength and irradiance (Figs. 7, 8). According to the results of the subgroup analysis, LLLT was more effective than the sham intervention, as indicated by changes in burning pain intensity. The meta-regression analysis showed only intervention frequency (regression coefficient: 1.263, 95% CI: 0.356 to 2.170, *P* = 0.006) was an influencing factor of the effect of LLLT on burning pain, while the risk of bias, publication year, laser wavelength, irradiance, and intervention duration showed no significant impact on it (Additional file 3).

### Secondary outcomes (quality of life)

**Fig. 9:**
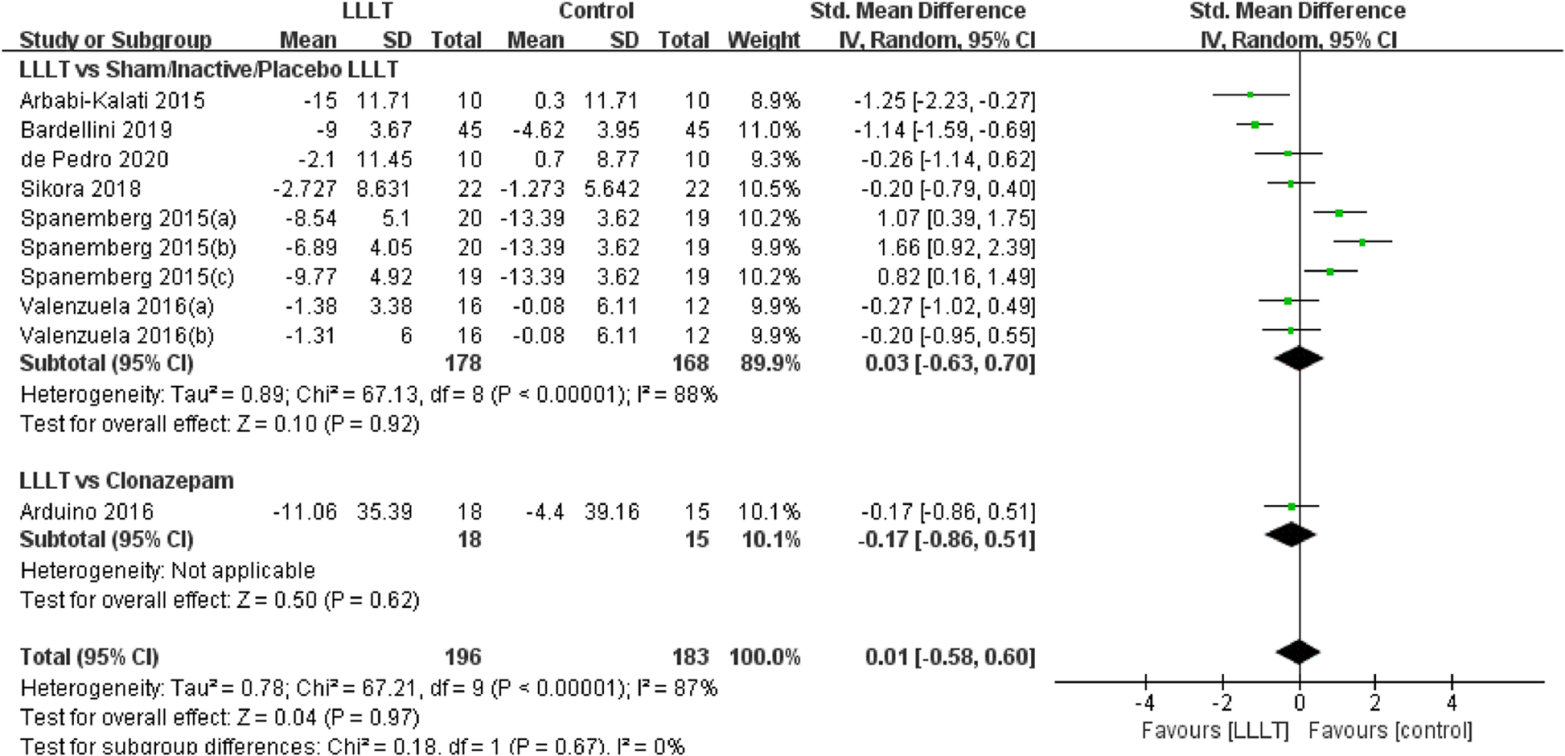
Forest plot and meta-analysis of changes in quality of life. Subgroup analysis according to different intervention methods

Changes in quality of life (measured by Oral Health Impact Profile-14) occurred in seven RCTs [20, 22, 24–26, 37, 40] involving 379 participants. Data evaluating the differences from baseline to final treatment evaluation for each study were extracted, and the pooled analysis revealed a statistically significant intergroup difference, along with a substantially high level of heterogeneity among the included studies. Additionally, no significant difference was observed when we performed a subgroup analysis for different interventions (SMD: 0.01, 95%CI: -0.58 to 0.60, *P* = 0.97; *I*^*2*^ = 87%; Fig. 9).

### Secondary outcomes (negative emotions)

**Fig. 10:**
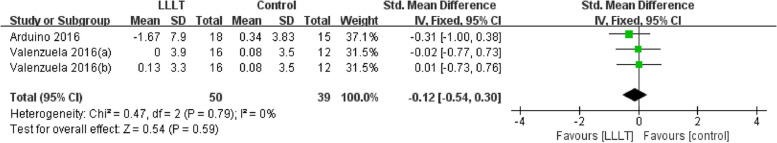
Differences in HADS scores (negative emotions) following LLLT compared with other forms of interventions

Negative emotions were reported in four RCTs; the HADS was used to measure anxiety and depression [22, 25, 39], the GDS was used to measure [22], and the SCL-90R was used to measure anxiety and depression [20]). Data extracted from a total of 89 patients were pooled to analyze the difference between baseline and final treatment evaluation for each study. The data favored the LLLT group, but no statistically significant intergroup differences were found among the pooled data (SMD: -0.12, 95% CI: -0.54 to 0.30, *P* = 0.59; *I*^*2*^ = 0%; Fig. 10), and there was a substantially low level of heterogeneity among the included studies.

### Secondary outcomes (other relevant outcomes and serious adverse events)

Salivary cortisol [23], TNF-α [27, 36], and IL-6 [36] were measured in three RCTs; oral salivary flow rate [22, 27] was examined in two RCTs; and the association between xerostomia and BMS [25] was investigated in one RCT. There were positive improvements in salivary cortisol [23] and IL-6 measures [36]. However, there were no significant improvements in TNF-α levels [27], salivary flow [22], and the association between xerostomia and BMS [25]. No serious adverse effects, such as worsening of symptoms, suicide, or death, were reported.

### Sensitivity analysis and publication bias

**Fig. 11:**
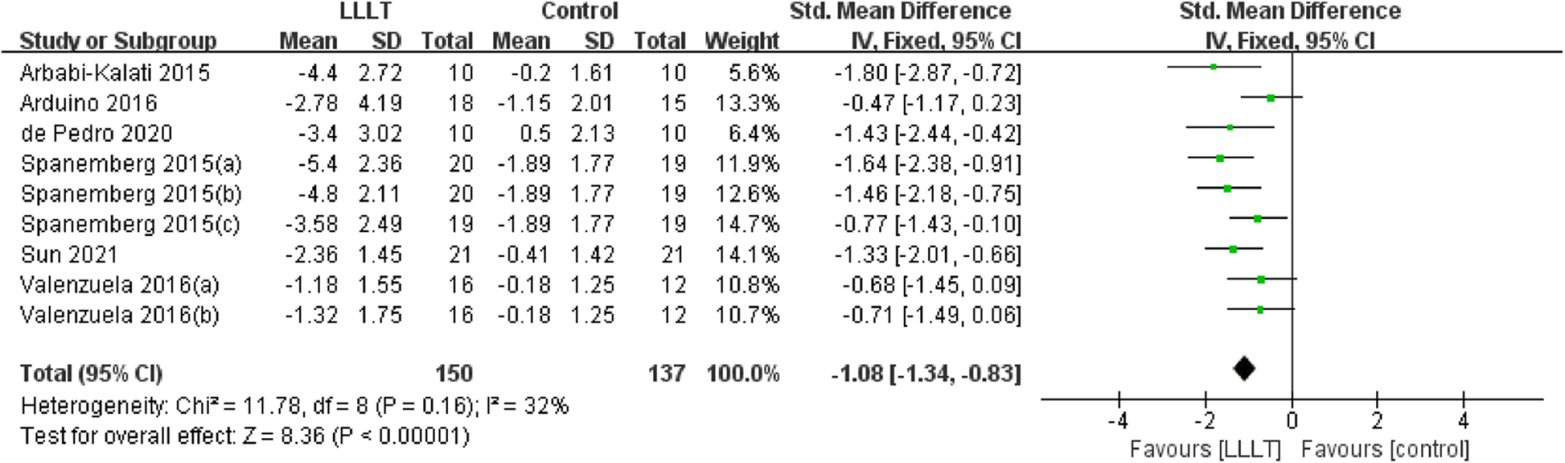
Sensitivity analysis for burning pain measured by the Visual Analog Scale. Forest plot and meta-analysis of changes in pain intensity after removing the studies of Sikora et al. and Skrinjar et al.

**Fig. 12:**
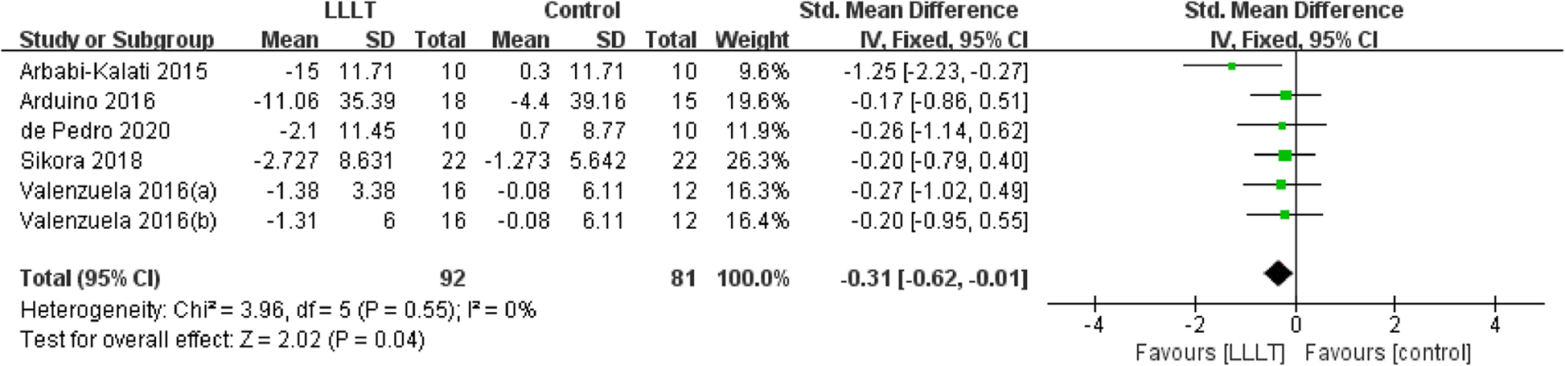
Sensitivity analysis for quality of life measured by the Oral Health Impact Profile-14. Forest plot and meta-analysis of changes in quality of life after removing the studies of Bardellini et al. and Spanemberg et al.

**Fig. 13:**
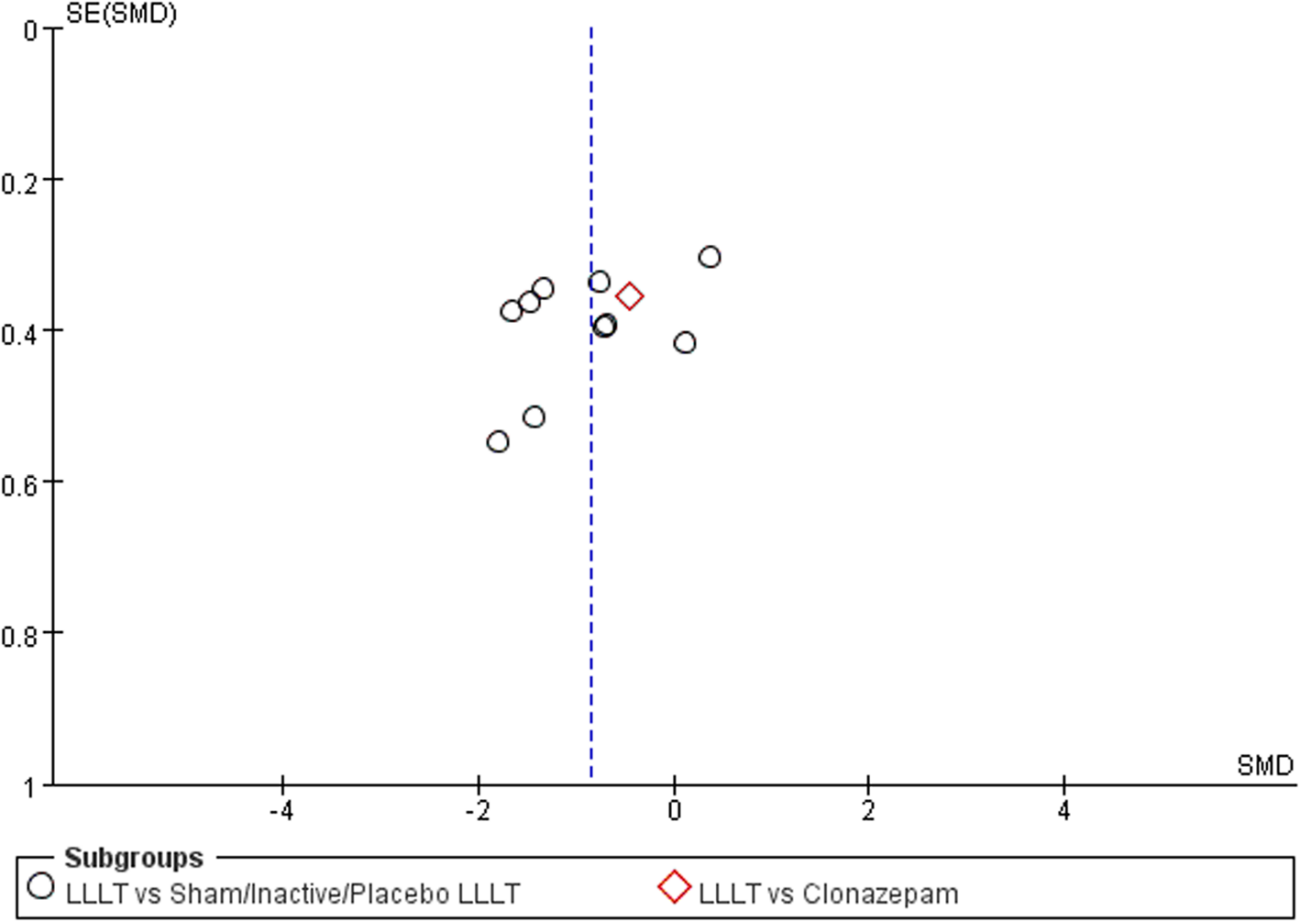
Funnel plot summary for outcomes before and after interventions (burning pain, measured by the Visual Analog Scale)

**Fig. 14:**
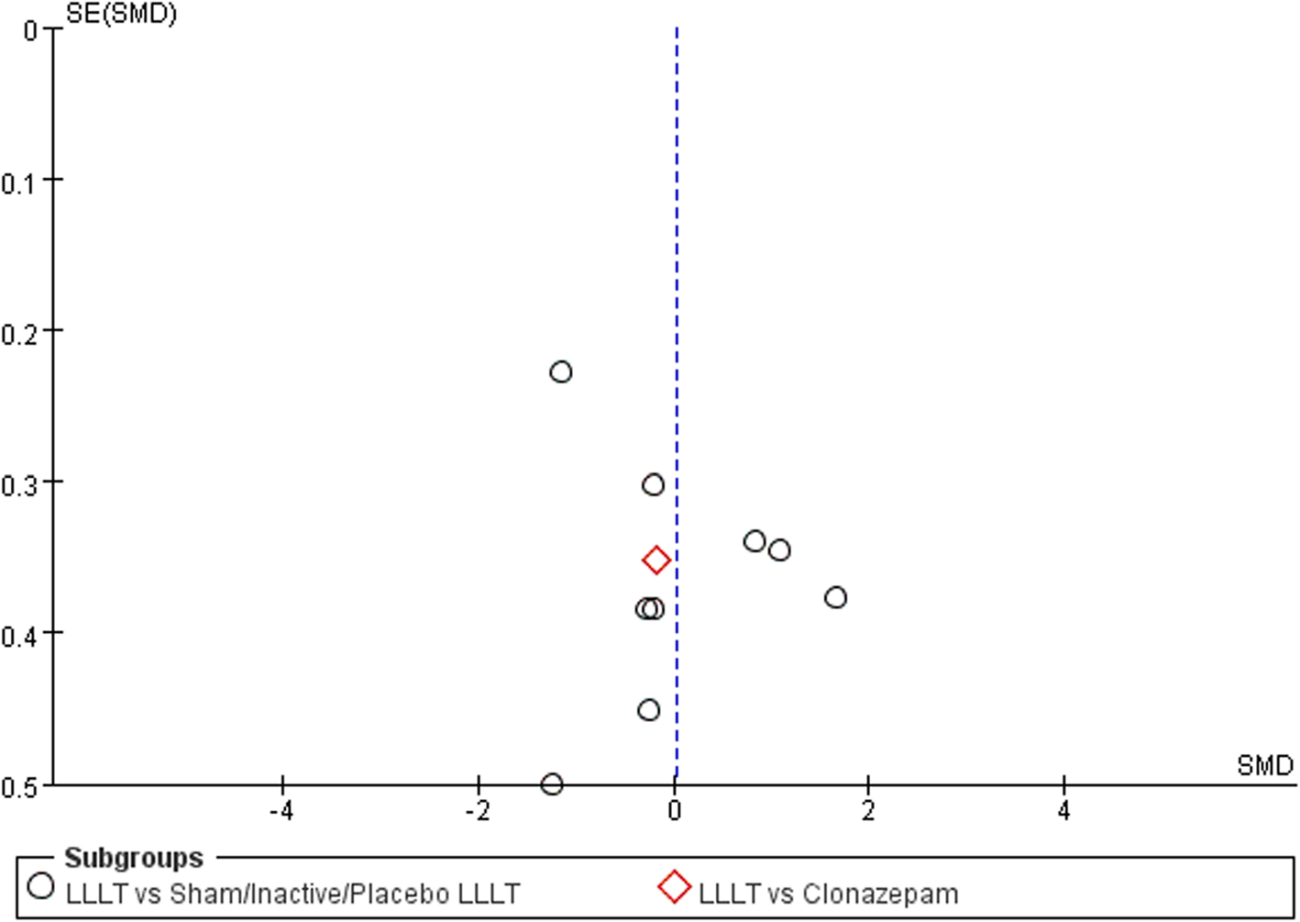
Funnel plot summary for outcomes before and after interventions (quality of life, measured by the Oral Health Impact Profile-14)

**Fig. 15:**
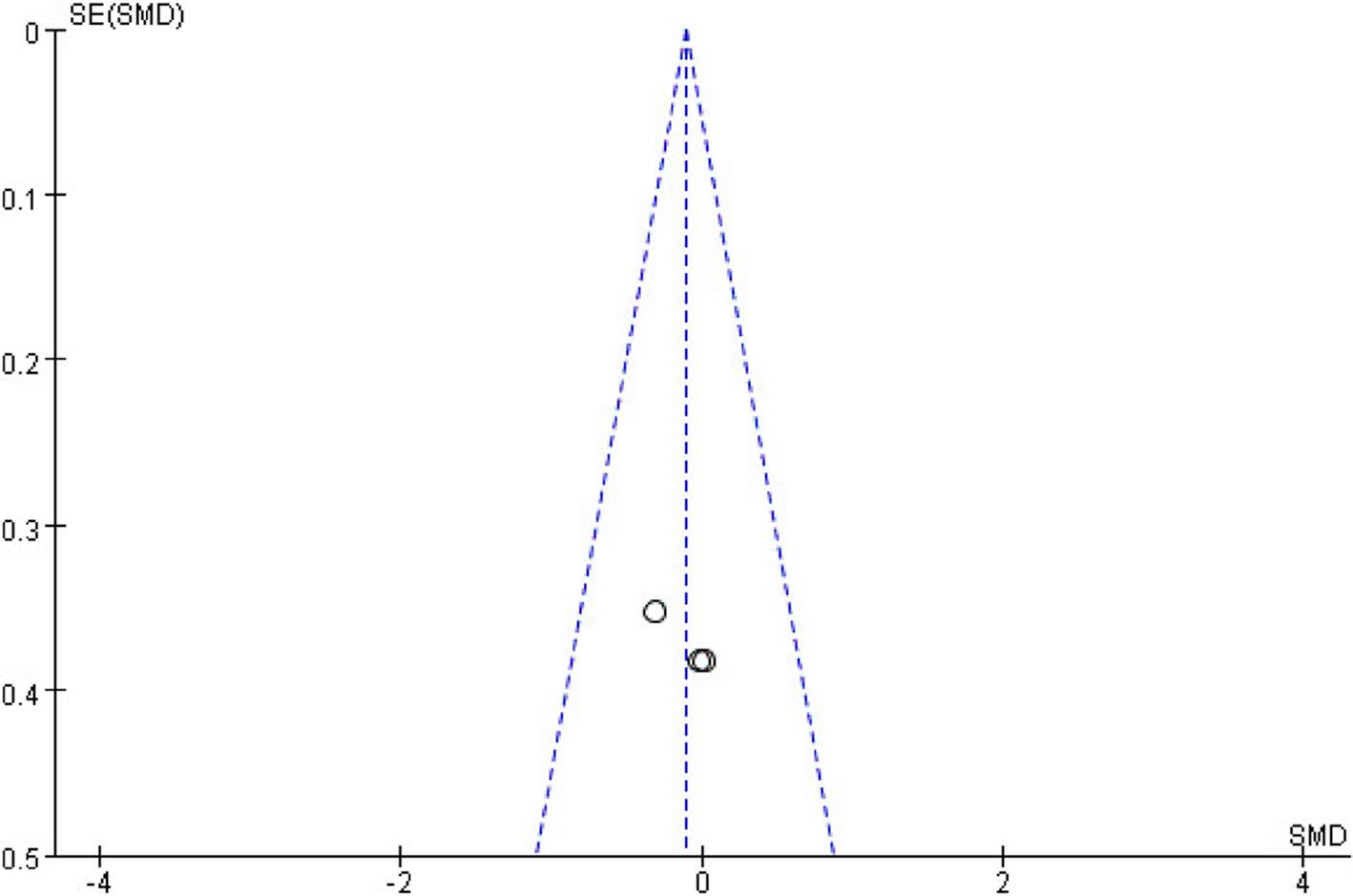
Funnel plot summary for outcomes before and after interventions (negative emotions, measured by the Hospital Anxiety and Depression Scale)

For pain intensity, sensitivity analysis showed that the studies by Sikora et al. [24] and Skrinjar et al. [23] may be the main cause of heterogeneity, as the *I*^2^ value decreased to 32% after these studies were removed (Fig. 11). In terms of quality of life, the studies by Bardellini et al. [40] and Spanemberg et al. [37] may be the main cause of heterogeneity according to the sensitivity analysis, as the *I*^2^ value decreased to 0% once these studies were removed (Fig. 12). The funnel plot of changes in pain intensity was symmetrical, meaning that no publication bias was detected (Fig. 13). The funnel plots for quality of life and anxiety were asymmetrical, thus indicating a significant risk of publication bias [42] (Figs. 14, 15).

## Discussion

LLLT is considered an important innovation in improving pain and therefore has great potential for therapeutic applications in neuropathic pain [43]. This meta-analysis found that LLLT (SMD: -0.87, 95% CI: -1.29 to -0.45) was more effective than sham LLLT or clonazepam in reducing burning pain without serious side effects. LLLT also had a positive effect on quality of life (SMD: 0.01, 95%CI: -0.58 to 0.60) and negative emotions (SMD: -0.12, 95% CI: -0.54 to 0.30), but these effects were not statistically significant.

Previous studies suggested that LLLT exerts potent anti-inflammatory effects in the peripheral nervous system and promote functional recovery and regeneration of peripheral nerves after injury [44]. The involvement of peripheral nerve fiber lesions in the sensory abnormalities and chronic pain mechanisms in the pathogenesis of BMS. Approximately 20% of patients with primary BMS developed trigeminal nerve damage involving primarily the lingual nerve, mandibular nerve, or the entire trigeminal nerve, and some studies have also found focal peripheral small nerve fiber lesions in the oral mucosa [45]. Lesions of small somatic nerve fibers could lead patients to experience burning pain, and numbness in the oral mucosa, usually more intense in the evening, while lesions of autonomic nerve fibers could make patients experience dry mouth [46], which is consistent with the disease characteristics of BMS (manifesting as mild pain in the morning and severe pain at night, usually accompanied by dry mouth symptoms). Proinflammatory cytokines, such as interleukin 1β (IL-1β), interleukin 2 (IL-2), IL-6, interleukin 8 (IL-8), and TNF-α, were found at higher levels in saliva or plasma in BMS patients, but anti-inflammatory cytokines, such as interleukin 10 (IL-10), were decreased [2, 47, 48].

This study found that the intervention frequency was an influencing factor of the effect of LLLT on burning pain. Consistent with previous systematic reviews, LLLT, 1 or 2 times per week, more than 4 weeks of intervention, was beneficial for reducing burning pain intensity in patients with BMS [49, 50]. This suggested that the effect of LLLT progresses over time and could maximize treatment results [51]. LLLT triggers a photochemical reaction in the cell rather than producing a thermal effect, a process also known as 'photobiomodulation' or 'photobiostimulation' [52]. The optical spectral range used in LLLT was between 600 and 1100 nm, which fell into an 'optical window' at red and near-infrared light wavelengths. Previous studies reported that longer wavelengths in the range of 780–950 nm, which penetrate further, were used to treat deeper-seated tissues, while wavelengths in the range of 600–700 nm were used to treat superficial tissues [53]. Our results indicated that wavelengths in these two spectral ranges have identical effectiveness in reducing burning pain. One possible explanation is that these wavelengths of LLLT influence the absorption and conversion efficiency of light energy by tissues or cells, improve the levels of inflammatory cytokines, promote recovery of nervous function, and thus show promising treatment success. After LLLT, the expression of these inflammatory cytokines (such as IL-1β, IL-6, IL-8, and TNF-α) significantly decreases to achieve a beneficial biomodulatory effect [54, 55]. Pezelj-Ribaric et al. [36] measured the levels of proinflammatory cytokines (TNF-α and IL-6) in whole unstimulated saliva in subjects with BMS before and after treatment with LLLT. The results revealed that the levels of TNF-α and IL-6 in the experimental group decreased after 4 weeks, accompanied by a slight improvement in burning sensation. The irradiance, another important influencing factor, may promote stimulation and healing at relatively low doses (5 to 50 mW/cm^2^), whereas higher doses (up to 50 mW/cm^2^) may be beneficial for nerve inhibition and pain relief [56]. Consistent with our results, most of the studies in this meta-analysis applied higher doses of irradiance. Relatively high doses of LLLT may reduce pain by inhibiting neural pathways for therapeutic purposes. From this perspective, high-dose irradiance may be a better choice for pain management in BMS patients. However, according to the results of the subgroup analysis, efficacy did not significantly differ by wavelength and irradiance.

Although current evidence suggests that LLLT can effectively reduce burning pain and numbness in BMS patients [12], it does not appear to improve BMS-induced xerostomia [25]. This lack of effect may be because LLLT improved the neural function of the small nerve fiber in the oral mucosa but not the function of the autonomic nerves that regulate saliva production [57]. This mechanism may also explain the reported improvements in burning pain and numbness [12], whereas salivary flow and BMS-induced xerostomia were not significantly improved [22, 25]. This hypothesis needs to be confirmed by further experimental research that examines the autonomic nervous system (ANS) as a potential treatment target to observe the improvement of salivary flow and BMS-induced xerostomia [58].

Spontaneous, persistent, or recurrent burning pain in the oral mucosa severely affects the quality of life of people with BMS. Zhang et al. [59] conducted a meta-analysis of seven groups in four trials [25, 26, 37, 40] and found that LLLT was effective in improving quality of life (MD, -3.43, 95% CI, -5.11 to -1.75) when compared to placebo LLLT. However, the findings of the current study showed that LLLT had a positive influence on the improvement of quality of life (SMD: 0.01; 95% CI: -0.58 to 0.60), but this improvement was not significant. Notably, the improvement in quality of life involved many different aspects, and LLLT may only affect burning pain. Improvement of quality of life may need prolonged and multidisciplinary interventions. Moreover, multidisciplinary therapy may be more effective in enhancing the quality of life than the current intervention method, which is excessively homogenous [60]. Therefore, multidisciplinary intervention designs, such as LLLT combined with functional movement, acupuncture, meditation, and psychological support, are recommended for future research on effectively improving the quality of life among patients with BMS [60–62].

The results of a quantitative assessment demonstrated that LLLT has a beneficial effect on negative emotions (SMD: -0.12, 95% CI: -0.54 to 0.30), which was consistent with a previous systematic review [63]. Accumulating evidence has revealed that dental anxiety, as a dispositional factor in dental situations, is associated with state anxiety and pain related to dental procedures [64], and studies have reported that depression and pain share biological pathways and neurotransmitters (serotonin (SE), norepinephrine (5-HT), dopamine (DA), and glutamate) [65]. Increased levels of peripheral proinflammatory cytokines and neuroinflammatory changes are also related to the physiopathology of depression and pain [66, 67] which also explains why the application of antidepressants (such as clonazepam and melatonin) can improve depression and burning pain [68]. LLLT can also be recommended for depressive disorder, anxiety disorder, and chronic pain [69]. This treatment may work by promoting functional recovery and regeneration and increasing levels of peripheral proinflammatory cytokines. A case–control series suggested that LLLT to the back and thighs may induce an antidepressant effect in patients with low back pain and concurrent depression [70]. We, therefore, speculated that relief of negative emotions in patients with BMS would be related to the clinically reduction in pain reported above.

### Limitations

The level evidence-based findings were low because of the lack of homogeneity of outcomes and long-term real-world efficacy data, which yielded results that did not provide strong evidence to the public. Subgroup analysis was used, and sensitivity analyses were performed by removing studies individually to examine the possible cause of heterogeneity among study results. Most studies we included had a common limitation, a small sample size and heterogeneity in study designs of LLLT protocols (including the wavelength, the irradiance, the intervention duration and the numbers of interventions). Publication bias cannot be completely ruled out, as we were not able to collect sufficient data from each study for each outcome. These limitations have been minimized by the comprehensive design and rigorous assessment of the data presented. To determine the ideal wavelength, irradiance, intervention duration and number of interventions, further large-sample trials are needed.

### Clinical implications

More high-quality studies on LLLT for patients with BMS are needed to enlarge the sample size and reduce bias. Longer follow-up trials are needed to observe the long-term effect of LLLT in the treatment of BMS. Multidisciplinary intervention is needed to observe the improvement in quality of life. No serious adverse effects have been reported after LLLT. A local burning sensation has been reported, but relief usually occurred within a few days. LLLT can be recommended as an alternative therapy when burning pain alone is not accompanied by dry mouth. The addition of a group of clinically and routinely used medications for comparison may be considered to increase the persuasiveness of the idea that LLLT is superior to or an alternative to drugs. To achieve the above requirements, a standardized trial design and a well-coordinated team are needed to help perform interventions successfully.

## Conclusions

Low-level laser therapy could reduce burning pain in patients with burning mouth syndrome, and have a positive influence on the quality of life and anxiety symptoms, without serious side effects, indicating that it may be an effective therapy for burning mouth syndrome. However, given the low methodological quality of the selected studies, our results should be interpreted with caution. A long-term course of intervention, a larger sample size, and a multidisciplinary intervention design are urgently needed.

### Supplementary Information

**Additional file 1.** Search strategies for the databases.

**Additional file 2.** Results of the GRADE assessment.

**Additional file 3.** The results of meta-regression of burning pain in patients with BMS.

## Data Availability

All data generated or analyzed during this study are included in this published article.

## Abbreviations

BMS: Burning mouth syndrome
LLLT: Low-level laser therapy
PRISMA: Preferred Reporting Items for Systematic Reviews and Meta-Analyses
PICOS: Population, interventions, comparisons, outcomes, study design
RCTs: Randomized controlled trials
VAS: Visual Analog Scale
OHIP-14: Oral Health Impact Profile-14
HADS: Hospital Anxiety and Depression Scale
GRADE: Grading of Recommendations, Assessment, Development and Evaluation
