# Supplementary material for: Effects of low-level laser therapy on burning pain and quality of life in patients with burning mouth syndrome: a systematic review and meta-analysis
Source: BMC Oral Health. 2023 Oct 9;23:734. doi: 10.1186/s12903-023-03441-w (PMC10561515; doi:10.1186/s12903-023-03441-w)
Supplement: Supplementary file 1 — Additional file 1. Search strategies for the databases. [file 12903_2023_3441_MOESM1_ESM.docx]

**Additional file 1 Search strategies for the databases.**

Search strategies for all databases Jan 22^th^, 2023

1. Search Strategy for PubMed: 34

#1 (Burning Mouth Syndromes OR Mouth Syndrome, Burning OR Mouth Syndromes, Burning OR Syndrome, Burning Mouth OR Syndromes, Burning Mouth) OR ("Burning Mouth Syndrome"[Mesh])

#2 "Low-Level Light Therapy"[Mesh] OR Light Therapies, Low-Level OR Light Therapy, Low-Level OR Low Level Light Therapy OR Low-Level Light Therapies OR Therapies, Low-Level Light OR Therapy, Low-Level Light OR Photobiomodulation Therapy OR Photobiomodulation Therapies OR Therapies, Photobiomodulation OR Therapy, Photobiomodulation OR LLLT OR Laser Therapy, Low-Level OR Laser Therapies, Low-Level OR Laser Therapy, Low Level OR Low-Level Laser Therapies OR Laser Irradiation, Low-Power OR Irradiation, Low-Power Laser OR Laser Irradiation, Low Power OR Low-Power Laser Therapy OR Low Power Laser Therapy OR Laser Therapy, Low-Power OR Laser Therapies, Low-Power OR Laser Therapy, Low Power OR Low-Power Laser Therapies OR Low-Level Laser Therapy OR Low Level Laser Therapy OR Low-Power Laser Irradiation OR Low Power Laser Irradiation OR Laser Biostimulation OR Biostimulation, Laser OR Laser Phototherapy OR Phototherapy, Laser

#3 #1 AND #2

#4 "Pain"[Mesh] OR Pain, Burning OR Burning Pain OR Burning Pains OR Pains, Burning OR Suffering, Physical OR Physical Suffering OR Physical Sufferings OR Sufferings, Physical OR Pain, Migratory OR Migratory Pain OR Migratory Pains OR Pains, Migratory OR Pain, Radiating OR Pains, Radiating OR Radiating Pain OR Radiating Pains OR Pain, Splitting OR Pains, Splitting OR Splitting Pain OR Splitting Pains OR Ache OR Aches OR Pain, Crushing OR Crushing Pain OR Crushing Pains OR Pains, Crushing

#5 Quality of Life"[Mesh] OR Life Quality OR Health-Related Quality Of Life OR Health Related Quality Of Life OR HRQOL

#6 #4 OR #5

#7 #3 AND #6

#8 "parallel"[Text Word] OR "controlled trial*"[Text Word] OR "random*"[Text Word] OR "randomi*"[Text Word] OR "intervention*"[Text Word] OR "clinical trial"[PT]

#9 animals NOT humans

#10 #8 OR #9

#11 #7 AND #10

2. Search Strategy for EMBASE: 29

#1 ‘Burning Mouth Syndrome*’ OR ‘Burning Mouth Syndrome’ OR ‘Mouth Syndrome, Burning’ OR ‘Mouth Syndromes, Burning’ OR ‘Syndrome, Burning Mouth’ OR ‘Syndromes, Burning Mouth’/exp OR ‘BMS’

#2 ‘Low-Level Light Therapy’/exp OR ‘LLLT’ OR ‘PBM’ OR ‘Low Level Light Therapy’ OR ‘Light Therapies, Low-Level’ OR ‘Light Therapy, Low-Level’ OR ‘Low-Level Light Therapies’ OR ‘Therapies, Low-Level Light’ OR ‘Therapy, Low-Level Light’ OR ‘Photobiomodulation Therapy’ OR ‘Photobiomodulation Therapies’ OR ‘Therapies, Photobiomodulation’ OR ‘Therapy, Photobiomodulation’ OR ‘Laser Therapy, Low-Level’ OR ‘Laser Therapies, Low-Level’ OR ‘Laser Therapy, Low Level’ OR ‘Low-Level Laser Therapies’ OR ‘Laser Irradiation, Low-Power’ OR ‘Irradiation, Low-Power Laser’ OR ‘Laser Irradiation, Low Power’ OR ‘Low-Power Laser Therapy’ OR ‘Low Power Laser Therapy’ OR ‘Laser Therapy, Low-Power’ OR ‘Laser Therapies, Low-Power’ OR ‘Laser Therapy, Low Power’ OR ‘Low-Level Laser Therapy’ OR ‘Low Level Laser Therapy’ OR ‘Low-Power Laser Irradiation’ OR ‘Low Power Laser Irradiation’ OR ‘Laser Biostimulation’ OR ‘Biostimulation, Laser’ OR ‘Laser Phototherapy’ OR ‘Phototherapy, Laser’

#3 #1 AND #2

#4 ‘Pain’/exp OR ‘Pain*’ OR ‘Ache*’ OR ‘Physical Suffering*’ OR ‘Suffering, Physical’ OR ‘Neck Pain’/exp OR ‘Cervicalgia*’ OR ‘Cervicodynia*’ OR ‘Neckache*’ OR ‘Headache’/exp OR ‘Headache*’ OR ‘Cephalodynia*’ OR ‘Cephalalgia*’ OR ‘Cephalgia*’

#5 ‘Quality of Life’/exp OR ‘Life Quality’ OR ‘Health-Related Quality Of Life’/exp OR ‘Health Related Quality Of Life’ OR ‘HRQOL’

#6 #4 OR #5

#7 #3 AND #6

#8 ‘Parallel’/exp OR ‘Observational’/exp OR ‘Cross-Sectional’/exp OR ‘Pre–Post’/exp OR ‘Before-After’/exp OR ‘Controlled Trial*’/exp OR ‘Random*’/exp OR ‘ Randomi*’/exp OR ‘Intervention*’/exp

#9 Random*:ab,ti OR Parallel:ab,ti OR Pre-Post:ab,ti OR Before-After:ab,ti

#10 #9 OR #10

#11  #7 AND #10

3. Search Strategy for Cochrane Library 53

#1 Burning Mouth Syndrome*:ti,ab,kw OR Burning Mouth Syndromes:ti,ab,kw OR BMS:ti,ab,kw OR Mouth Syndrome, Burning:ti,ab,kw OR Mouth Syndromes, Burning:ti,ab,kw OR Syndrome, Burning Mouth:ti,ab,kw OR Syndromes, Burning Mouth:ti,ab,kw OR Burning Mouth Syndrome:ti,ab,kw

#2 Low-Level Light Therapy:ti,ab,kw OR LLLT:ti,ab,kw OR PBM:ti,ab,kw OR Light Therapies, Low-Level:ti,ab,kw OR Light Therapy, Low-Level:ti,ab,kw OR Low Level Light Therapy:ti,ab,kw OR Low-Level Light Therapies:ti,ab,kw OR Therapies, Low-Level Light:ti,ab,kw OR Therapy, Low-Level Light:ti,ab,kw OR Photobiomodulation Therapy:ti,ab,kw OR Photobiomodulation Therapies:ti,ab,kw OR Therapies, Photobiomodulation:ti,ab,kw OR Therapy, Photobiomodulation:ti,ab,kw OR LLLT:ti,ab,kw OR Laser Therapy, Low-Level:ti,ab,kw OR Laser Therapies, Low-Level:ti,ab,kw OR Laser Therapy, Low Level:ti,ab,kw OR Low-Level Laser Therapies:ti,ab,kw OR Laser Irradiation, Low-Power:ti,ab,kw OR Irradiation, Low-Power Laser:ti,ab,kw OR Laser Irradiation, Low Power:ti,ab,kw OR Low-Power Laser Therapy:ti,ab,kw OR Low Power Laser Therapy:ti,ab,kw OR Laser Therapy, Low-Power:ti,ab,kw OR Laser Therapies, Low-Power:ti,ab,kw OR Laser Therapy, Low Power:ti,ab,kw OR Low-Power Laser Therapies:ti,ab,kw OR Low-Level Laser Therapy:ti,ab,kw OR Low Level Laser Therapy:ti,ab,kw OR Low-Power Laser Irradiation:ti,ab,kw OR Low Power Laser Irradiation:ti,ab,kw OR Laser Biostimulation:ti,ab,kw OR Biostimulation, Laser:ti,ab,kw OR Laser Phototherapy:ti,ab,kw OR Phototherapy, Laser:ti,ab,kw

#3 #1 AND #2

#4 Pain*:ti,ab,kw OR Ache*:ti,ab,kw OR Physical Suffering*:ti,ab,kw OR Cervicalgia*:ti,ab,kw OR Cervicodynia*:ti,ab,kw OR Neckache*:ti,ab,kw OR Headache*:ti,ab,kw OR Cephalodynia*:ti,ab,kw OR Cephalalgia*:ti,ab,kw OR Cephalgia*:ti,ab,kw

#5 Quality of Life:ti,ab,kw OR Life Quality:ti,ab,kw OR Health-Related Quality Of Life:ti,ab,kw OR Health Related Quality Of Life:ti,ab,kw OR HRQOL:ti,ab,kw

#6 #4 OR #5

#7 #3 AND #6

#8 Parallel OR Pre-Post OR Before-After OR Controlled Trial* OR Random* OR Randomi* OR Intervention*

#9 Randomized Controlled Trial:pt OR Clinical Trial:pt

#10 #8 OR #9

#11 #7 AND #10

4. Search Strategy for Web of Science 24

#1 TS=(“Burning Mouth Syndrome*” OR “Burning Mouth Syndrome” OR “BMS” OR “Mouth Syndrome, Burning” OR “Mouth Syndromes, Burning” OR “Syndrome, Burning Mouth” OR “Syndromes, Burning Mouth”)

#2 TS=(“Low-Level Light Therapy” OR “LLLT” OR “PBM” OR “Low Level Light Therapy” OR “Light Therapies, Low-Level” OR “Light Therapy, Low-Level” OR “Low-Level Light Therapies” OR “Therapies, Low-Level Light” OR “Therapy, Low-Level Light” OR “Photobiomodulation Therapy” OR “Photobiomodulation Therapies” OR “Therapies, Photobiomodulation” OR “Therapy, Photobiomodulation” OR “Laser Therapy, Low-Level” OR “Laser Therapies, Low-Level” OR “Laser Therapy, Low Level” OR “Low-Level Laser Therapies” OR “Laser Irradiation, Low-Power” OR “Irradiation, Low-Power Laser” OR “Laser Irradiation, Low Power” OR “Low-Power Laser Therapy” OR “Low Power Laser Therapy” OR “Laser Therapy, Low-Power” OR “Laser Therapies, Low-Power” OR “Laser Therapy, Low Power” OR “Low-Level Laser Therapy” OR “Low Level Laser Therapy” OR “Low-Power Laser Irradiation” OR “Low Power Laser Irradiation” OR “Laser Biostimulation” OR “Biostimulation, Laser” OR “Laser Phototherapy” OR “Phototherapy, Laser”)

#3 #1 AND #2

#4 TS=(“Pain*” OR “Ache*” OR “Physical Suffering*” OR “Neckache*” OR “Cervicalgia*” OR “Cervicodynia*” OR “Headache*” OR “Cephalodynia*” OR “Cephalalgia*” OR “Cephalgia*”)

#5 TS=(“Quality of Life”/exp OR “Life Quality” OR “Health-Related Quality Of Life”/exp OR “Health Related Quality Of Life” OR “HRQOL”)

#6 #4 OR #5

#7 TS=(parallel OR observational OR cross-sectional OR pre–post OR before-after OR controlled trial* OR random* OR randomi* OR intervention*)

#8 #3 AND #6 AND #7

5. Search Strategy for CINAHL (Ebsco) 79

S1 TX (“Burning Mouth Syndrome*” OR “Burning Mouth Syndrome” OR “BMS” OR “Mouth Syndrome, Burning” OR “Mouth Syndromes, Burning” OR “Syndrome, Burning Mouth” OR “Syndromes, Burning Mouth”)

S2 TX (“Low-Level Light Therapy” OR “LLLT” OR “PBM” OR “Low Level Light Therapy” OR “Light Therapies, Low-Level” OR “Light Therapy, Low-Level” OR “Low-Level Light Therapies” OR “Therapies, Low-Level Light” OR “Therapy, Low-Level Light” OR “Photobiomodulation Therapy” OR “Photobiomodulation Therapies” OR “Therapies, Photobiomodulation” OR “Therapy, Photobiomodulation” OR “Laser Therapy, Low-Level” OR “Laser Therapies, Low-Level” OR “Laser Therapy, Low Level” OR “Low-Level Laser Therapies” OR “Laser Irradiation, Low-Power” OR “Irradiation, Low-Power Laser” OR “Laser Irradiation, Low Power” OR “Low-Power Laser Therapy” OR “Low Power Laser Therapy” OR “Laser Therapy, Low-Power” OR “Laser Therapies, Low-Power” OR “Laser Therapy, Low Power” OR “Low-Level Laser Therapy” OR “Low Level Laser Therapy” OR “Low-Power Laser Irradiation” OR “Low Power Laser Irradiation” OR “Laser Biostimulation” OR “Biostimulation, Laser” OR “Laser Phototherapy” OR “Phototherapy, Laser”)

S3 S1 AND S2

S4 TX (“Pain*” OR “Ache*” OR “Acute Pain*” OR “Physical Suffering*” OR “Suffering, Physical” OR “Cervicalgia*” OR “Cervicodynia*” OR “Neckache*” OR “Cephalodynia*” OR “Cephalalgia*” OR “Cephalgia*”)

S5 TX (“Quality of Life”/exp OR “Life Quality” OR “Health-Related Quality Of Life”/exp OR “Health Related Quality Of Life” OR “HRQOL”)

S6 S4 OR S5

S7 MH (“random assignment” OR “placebos” OR “placebo effect” OR “single-blind studies” OR “double-blind studies” OR “triple-blind studies” OR “randomized controlled trials” OR “comparative studies” OR “evaluation research” OR “prospective studies” OR “crossover design” OR “prospective studies” OR “clinical trials” OR “clinical trial registry”)

S8 TX (random* OR allocation OR placebo* OR “single blind” OR “double blind” OR “comparative study” OR “evaluation stud*” OR “follow-up stud*” OR “prospective stud*” OR “cross-over stud*” OR control* OR prospective* OR volunteer* OR “RCT” OR “clinical trial*”)

S9 PT (“randomized controlled trial” OR “clinical trial”)

S10 S7 OR S8 OR S9

S11 S3 AND S6 AND S10

6. Search Strategy for Scopus 35

1 TITLE-ABS-KEY (“Burning Mouth Syndrome*” OR “Burning Mouth Syndrome” OR “BMS” OR “Mouth Syndrome, Burning” OR “Mouth Syndromes, Burning” OR “Syndrome, Burning Mouth” OR “Syndromes, Burning Mouth”)

2 TITLE-ABS-KEY (“Low-Level Light Therapy” OR “LLLT” OR “PBM” OR “Low Level Light Therapy” OR “Light Therapies, Low-Level” OR “Light Therapy, Low-Level” OR “Low-Level Light Therapies” OR “Therapies, Low-Level Light” OR “Therapy, Low-Level Light” OR “Photobiomodulation Therapy” OR “Photobiomodulation Therapies” OR “Therapies, Photobiomodulation” OR “Therapy, Photobiomodulation” OR “Laser Therapy, Low-Level” OR “Laser Therapies, Low-Level” OR “Laser Therapy, Low Level” OR “Low-Level Laser Therapies” OR “Laser Irradiation, Low-Power” OR “Irradiation, Low-Power Laser” OR “Laser Irradiation, Low Power” OR “Low-Power Laser Therapy” OR “Low Power Laser Therapy” OR “Laser Therapy, Low-Power” OR “Laser Therapies, Low-Power” OR “Laser Therapy, Low Power” OR “Low-Level Laser Therapy” OR “Low Level Laser Therapy” OR “Low-Power Laser Irradiation” OR “Low Power Laser Irradiation” OR “Laser Biostimulation” OR “Biostimulation, Laser” OR “Laser Phototherapy” OR “Phototherapy, Laser”)

3 1 AND 2

4 TITLE-ABS-KEY (“Pain*” OR “Ache*” OR “Acute Pain*” OR “Physical Suffering*” OR “Suffering, Physical” OR “Cervicalgia*” OR “Cervicodynia*” OR “Neckache*” OR “Cephalodynia*” OR “Cephalalgia*” OR “Cephalgia*”)

5 TITLE-ABS-KEY (“Quality of Life” OR “Life Quality” OR “Health-Related Quality Of Life” OR “Health Related Quality Of Life” OR “HRQOL”)

6 4 OR 5

7 TITLE-ABS-KEY (“random assignment” OR “placebos” OR “placebo effect” OR “single-blind studies” OR “double-blind studies” OR “triple-blind studies” OR “randomized controlled trials” OR “comparative studies” OR “evaluation research” OR “prospective studies” OR “crossover design” OR “prospective studies” OR “clinical trials” OR “clinical trial registry”)

8 TITLE-ABS-KEY (random* OR allocation OR placebo* OR “single blind” OR “double blind” OR “comparative study” OR “evaluation stud*” OR “follow-up stud*” OR “prospective stud*” OR “cross-over stud*” OR control* OR prospective* OR volunteer* OR “RCT” OR “clinical trial*”)

9 TITLE-ABS-KEY (“randomized controlled trial” OR “clinical trial”)

10 7 OR 8 OR 9

11 3 AND 6 AND 10
