# Supplementary material for: Effects of low-level laser therapy on burning pain and quality of life in patients with burning mouth syndrome: a systematic review and meta-analysis
Source: BMC Oral Health. 2023 Oct 9;23:734. doi: 10.1186/s12903-023-03441-w (PMC10561515; doi:10.1186/s12903-023-03441-w)
Supplement: Supplementary file 2 — Additional file 2. Results of the GRADE assessment. [file 12903_2023_3441_MOESM2_ESM.docx]

**Additional file 2 Results of the GRADE assessment.**

| **Effects of Low-Level Laser Therapy on Burning Pain and Quality of Life in Patients with Burning Mouth Syndrome: A Systematic Review and Meta-Analysis** | | | | | | |
| --- | --- | --- | --- | --- | --- | --- |
| **Patient or population:** Patients with burning mouth syndrome. **Settings:** Clinical. **Intervention:** Low-level laser therapy.  **Comparison:** No treatment or other treatments. | | | | | | |
| **Outcomes** | **Illustrative comparative risks* (95% CI)** | | **Relative effect (95% CI)** | **No of Participants (studies)** | **Quality of the evidence (GRADE)** | **Comments** |
|  | Assumed risk | Corresponding risk |  |  |  |  |
|  | **Control** | **Low-Level Laser Therapy** |  |  |  |  |
| **Pain/Burning sensation**  Visual analogue scale (VAS)  Duration: median 4 weeks  Follow-up: median 8 weeks |  | The mean pain/burning sensation in the intervention groups was **0.87 standard deviations lower** (1.29 lower to 0.45 lower) |  | 354 (8 studies) | ⊕⊝⊝⊝ **very low**^1,2,3,4^ | SMD -0.87 (-1.29 to -0.45) |
| **Quality of life**  The oral health impact profile-14 (OHIP-14)  Duration: median 5 weeks  Follow-up: median 8 weeks |  | The mean quality of life in the intervention groups was **0.01 standard deviations higher** (0.58 lower to 0.60 higher) |  | 379 (7 studies) | ⊕⊝⊝⊝ **very low**^1,2,3,4^ | SMD 0.01 (-0.58 to 0.60) |
| *The basis for the **assumed risk** (e.g. the median control group risk across studies) is provided in footnotes. The **corresponding risk** (and its 95% confidence interval) is based on the assumed risk in the comparison group and the **relative effect** of the intervention (and its 95% CI). **CI:** Confidence interval; | | | | | | |
| GRADE Working Group grades of evidence **High quality:** Further research is very unlikely to change our confidence in the estimate of effect.  **Moderate quality:** Further research is likely to have an important impact on our confidence in the estimate of effect and may change the estimate. **Low quality:** Further research is very likely to have an important impact on our confidence in the estimate of effect and is likely to change the estimate. **Very low quality:** We are very uncertain about the estimate. | | | | | | |
| ^1^ >25% of the participants was from studies with high risk of bias. ^2^ I^2^ > 50%. ^3^ Publication bias was shown in funnel plots. ^4^ There were less than 400 participants in total. | | | | | | |
