# Supplementary material for: Effects of low-level laser therapy on burning pain and quality of life in patients with burning mouth syndrome: a systematic review and meta-analysis
Source: BMC Oral Health. 2023 Oct 9;23:734. doi: 10.1186/s12903-023-03441-w (PMC10561515; doi:10.1186/s12903-023-03441-w)
Supplement: Supplementary file 3 — Additional file 3. The results of meta-regression of burning pain in patients with BMS. [file 12903_2023_3441_MOESM3_ESM.doc]

**Additional file 3 The results of meta-regression of burning pain in patients with BMS.**

| **Variables** | **Burning pain** | | | | | | |
| --- | --- | --- | --- | --- | --- | --- | --- |
|  | | **Coefficient** | **SE** | **95% lower CI** | **95% upper CI** | **Z-value** | **P value** |
| Risk of bias | | 0.721 | 1.031 | -1.299 | 2.742 | 0.70 | 0.484 |
| Publication year | | 0.154 | 0.538 | -0.900 | 1.208 | 0.29 | 0.775 |
| Laser wavelength | | 0.014 | 0.606 | -1.174 | 1.203 | 0.02 | 0.981 |
| Laser irradiance | | -0.649 | 0.771 | -2.160 | 0.862 | -0.84 | 0.400 |
| Intervention duration | | 0.234 | 1.716 | -3.129 | 3.597 | 0.14 | 0.892 |
| Intervention frequency | | 1.263 | 0.463 | 0.356 | 2.170 | 2.73 | 0.006 |

Risk of bias (high, low, unclear risk of bias), publication year (< 5 years, > or = 5 years), laser wavelength (> 780 nm, 600-700 nm), irradiance (> 50 mW/cm^2^, < or = 50 mW/cm^2^), intervention duration (< or = 4 weeks intervention, > 4 weeks intervention), and intervention frequency (< or = 2 times intervention per week, > 2 times intervention per week).
